# A Biomimetic Framework for Collective Sensing and Immune-Inspired Verification in Complex Risk Analysis

**DOI:** 10.3390/biomimetics11060371

**Published:** 2026-05-27

**Authors:** Wei Meng

**Affiliations:** 1 International College, Dhurakij Pundit University, Bangkok 10210, Thailand; wei.men@dpu.ac.th; 2 The University of Western Australia, Perth 6009, Australia

**Keywords:** complex risk-information analysis, biomimetic collective sensing, immune-inspired verification, multi-objective optimization, governance auditing

## Abstract

Generative AI, retrieval-augmented architectures, and multi-source automated analytical tools are now being deployed in increasingly exacting risk-analytic environments. Yet faster processing has not yielded commensurate reductions in false alarms, missed alarms, hallucinated outputs, or failures of responsibility attribution. Against that background, this study develops a biomimetic framework that integrates collective sensing with immune-inspired verification for analyzing complex risk information. Using an openly documented two-layer data architecture that combines authentic public-source samples with rule-generated, synthetic derivative samples, the study links biological-to-engineering mechanism translation, multi-objective optimization, National Institute of Standards and Technology (NIST)-aligned evaluation, and a governance-compatibility index within a single auditable design chain. The present evidence indicates that risk level continues to show a stable positive association with threat scores. At the same time, fabricated relations, despite their smaller aggregate volume, are more likely to accumulate in high-risk intervals. These patterns suggest that structural perturbations are more consequential than mere high-frequency noise in distorting judgment. More importantly, the study establishes the empirical and methodological conditions for a formal comparison of recognition quality, system resilience, and governance compatibility. Taken together, the paper offers a testable biomimetic mechanism model and a reproducible evaluative blueprint for auditable optimization in complex risk-information analysis.

## 1. Introduction

Generative AI, retrieval-augmented systems, and multi-source automated analytical tools are increasingly being deployed in complex risk-information analysis settings, enabling organizations to complete signal filtering, pattern recognition, and risk stratification more rapidly under conditions of high noise, cross-linguality, multimodality, and adversarial input. Yet greater speed has not translated reliably into better judgment. False alarms, missed alarms, hallucinated outputs, distorted explanations, and misplaced trust expose engineering vulnerabilities that can be analyzed as problems of recognition and collaborative governance [1]. The real challenge, therefore, is not merely one of technological deployment, but a theoretical and methodological question of how to preserve recognition quality, system resilience, human–machine collaboration, and accountability simultaneously in complex environments.

This study is confined to defensive identification, verification, and governance-audit contexts. In this study, a complex risk-information analysis system is defined as a class of systems that identify, verify, and stratify multi-source, multimodal, time-varying information while supporting decision-making under potential adversarial perturbations. Its core task is not single-shot classification, but the continuous integration of evidence and risk judgment under uncertainty. The proposed biomimetic framework extracts transferable mechanisms from distributed vigilance in collective organisms and hierarchical recognition in immune systems, translating them into engineering variables, algorithmic rules, and auditable decision pathways. Its contribution to biomimetics lies in integrating the distributed early-warning mechanisms of collective vigilance and the hierarchical recognition–memory mechanisms of immune systems into an auditable optimization framework for analyzing complex risk information, thereby extending the domain of biomimetic intelligent computation to high-noise, multi-source, and multimodal decision settings.

Three interrelated research traditions broadly frame the problem. The first focuses on the enabling role of artificial intelligence in complex analytic settings and generally agrees that AI can improve large-scale data triage, pattern retrieval, and initial warning efficiency, while also emphasizing that bias, hallucination, and automation bias in high-risk settings cannot be ignored [1,2]. The second focuses on swarm intelligence, highlighting the effectiveness of distributed perception, local interaction, and global optimization in solving complex problems; foundational and computational studies indicate that swarm intelligence exhibits strong adaptability in optimization and search tasks [3,4]. The third focuses on artificial immune systems, emphasizing the potential of self/non-self discrimination, anomaly detection, and memory updating in complex detection tasks. However, their evaluation standards and external applicability boundaries are not always stable [5,6].

Nevertheless, important disagreements and deficiencies remain. On the one hand, research on explanation and trust is inconsistent: one line of argument maintains that explainability helps improve understanding and appropriate reliance, whereas another warns that under time pressure and high complexity, explanations themselves may induce excessive acceptance of system outputs and thereby amplify automation bias [1,2]. On the other hand, swarm intelligence research tends to center on global optimization performance, whereas artificial immune system research centers on anomaly detection; the two are seldom integrated into a unified mechanism chain of “early discovery–hierarchical verification–error-memory correction–responsibility retention.” Moreover, most evidence derives from standard optimization benchmarks, isolated anomaly-detection settings, or specific technical modules. It thus has not clarified whether these conclusions still hold in complex risk information analysis environments characterized by high noise, multi-source inputs, multimodality, and pressure from human–machine collaboration.

Building on this dialogue in the literature, the study specifies three gaps. First, existing research has not clearly explained why false alarms, missed alarms, hallucinations, and misplaced trust emerge jointly when multi-source fusion, generative inference, and human review coexist in complex risk information analysis systems. This first gap is mechanistic, as objects, processes, and consequences have not been incorporated into a single analytical chain. Second, existing research has not adequately tested whether distributed perception and threshold propagation in collective vigilance, together with hierarchical recognition and memory updating in immune systems, can generate additive rather than mutually offsetting optimization effects within the same system. The second gap is relational, because the direction, conditions, and strength of interaction among mechanisms remain unclear. Third, existing research has not clarified under what conditions these mechanisms improve recognition quality, system resilience, and trust calibration, and under what conditions their gains attenuate. The third is a boundary problem, as extrapolation under high-noise, multimodal, and time-pressured conditions remains constrained. In response, this study translates collective vigilance and immune recognition into a unified, biomimetic, collective-sensing–immune-inspired verification framework for analyzing complex risk information. This framework is evaluated through multiple lines of evidence spanning recognition performance, behavioral response, and governance compatibility.

In light of these gaps, the objective of this study is to develop and examine “Collective Vigilance Intelligence: A Biomimetic Framework for Collective Sensing and Immune-Inspired Verification in Complex Risk Analysis,” thereby answering three research questions. First, can complex risk information analysis systems characterized by high noise, multi-source inputs, multimodality, and adversarial inputs, with distributed collective sensing and threshold propagation mechanisms, relative to non-biomimetic baselines, stably improve recognition quality and early-discovery capability, as assessed by accuracy, *F*_1_, false-escalation rate (FER), missed-detection rate, and recovery rate? Second, in comparable systems, can immune-style rapid screening, deep verification, and memory-updating mechanisms improve system resilience and its performance recovery trajectory under perturbation by suppressing error propagation and strengthening response upon re-encounter? Third, in complex risk information analysis systems operating under explicit governance constraints and retaining human final adjudication, can biomimetic verification mechanisms significantly improve governance compatibility indicators by enhancing transparency, traceability, bias sensitivity, and the clarity of responsibility mapping? Although alternative mechanisms may exist, such as model scale, retrieval quality, or source coverage differences, the study distinguishes among them through multi-baseline comparisons, ablation experiments, robustness tests, and governance audits. These questions are examined through three lines of evidence: recognition performance, recovery under perturbation, and audit-log analysis.

The study is expected to contribute at three levels. Theoretically, it advances collective vigilance and immune recognition by integrating separate biomimetic metaphors into complementary modules within a single mechanism chain, thereby offering a more testable explanatory framework for biomimetic information processing in complex risk analysis. Methodologically, it constructs a multi-objective optimization design that simultaneously accommodates recognition performance, system resilience, and governance compatibility, and strengthens reviewability through multiple baselines, ablations, robustness testing, and governance audits. In practice, it aims to provide an operational, modular framework for system design and audit governance in high-noise, multi-source risk analysis settings. Its distinctive contribution lies not in invoking biological analogy as a rhetorical device but in converting distributed vigilance and immune-style verification into a single auditable mechanism chain that can be evaluated, challenged, and refined within governance-sensitive analytical environments. The conclusions primarily apply to open-source, multi-source, multimodal complex risk information analysis settings in which human final adjudication is retained; they are not extrapolated to all high-risk automated systems, and any extension beyond this scope requires caution. At the same time, the systems discussed here are restricted to defensive identification, verification, and governance-audit settings and do not involve the optimization of real-world attack execution pathways.

The distinctive contribution of this study lies not in borrowing biological metaphors as decorative analogy, but in preserving biological function at the level of mechanism. It converts distributed vigilance and immune-style verification into a single auditable chain of local sensing, threshold propagation, layered verification, memory retention, and responsibility mapping, thereby giving biomimetic optimization a firmer epistemic, methodological, and governance-sensitive footing.

The remainder of the paper is organized as follows. Section 2 reviews the relevant literature and refines the theoretical foundation. Section 3 presents the research method and design, including biological-mechanism mapping, multi-objective optimization settings, data sources, synthetic data generation, reproducibility protocols, and the governance compatibility index. Section 4 reports the main experiment, ablation experiments, robustness tests, and governance-audit results. Section 5 discusses theoretical implications, governance implications, boundary conditions, and alternative explanations. Section 6 concludes and outlines directions for future research.

## 2. Literature Review

Research on complex risk-information analysis systems is moving beyond processing speed alone toward a broader concern with robustness, collaborative reliability, and governance accountability. Generative AI, retrieval-augmented systems, and multi-source automated analytical tools have substantially expanded the scale and speed of information filtering, pattern recognition, and early warning. Yet false alarms, missed alarms, hallucinated outputs, distorted explanations, and misplaced trust have not disappeared accordingly. Related studies indicate that automation-enabled gains and system vulnerabilities often increase in parallel, especially under conditions of high noise, cross-linguality, multimodality, and adversarial input [1,7]. Accordingly, complex risk-information analysis is not merely a matter of improving performance; it is a composite problem involving recognition quality, system resilience, human–machine collaboration, and governance compatibility.

This review structures the literature into five interrelated strands: enablement and distortion in complex risk-information analysis; the biomimetic logic of collective vigilance and distributed collective sensing; hierarchical verification and memory updating in artificial immune systems; the role of explainability and trust calibration in human–machine collaboration; and institutional constraints relating to governance, auditing, and dual-use boundaries. It follows a progressive analytical path of “concept–mechanism–method–boundary–governance,” thereby allowing the subsequent research questions, research design, and evaluation system to be developed and tested within a single logical framework.

The first strand examines automated analysis as an enabling capability in complex information environments. Studies are fairly consistent in finding that automated analysis can improve large-scale data triage, pattern retrieval, and the initial warning efficiency, thereby enhancing early discovery in complex environments [1]. However, security research on multimodal models also shows that textual, speech, and image channels are not independent sources of risk; rather, they may jointly constitute composite pathways for adversarial attacks and error propagation [7]. This suggests that, in complex risk-information analysis systems, automation does not necessarily yield a corresponding increase in reliability.

The main limitation of this literature is that it is better at identifying where failures occur than at explaining why multiple failures occur within the same workflow. Methodologically, many studies rely on single-module security evaluations, local adversarial tests, or task-specific experiments, which can easily overestimate a model’s transferability to real workflows. Theoretically, the literature often discusses hallucination, adversarial perturbation, retrieval mismatch, or automation bias in isolation, rather than situating them within a unified chain of complex risk-information analysis. Boundary conditions are likewise unclear: robustness conclusions derived from standard benchmark tasks cannot necessarily be extrapolated directly to complex risk-information analysis settings that retain human final adjudication and operate under intense time pressure. A plausible competing explanation is that observed failures may stem less from the absence of biomimetic mechanisms than from data bias, retrieval quality, or model-scale differences; existing research remains insufficient to systematically distinguish these explanations.

The second strand focuses on swarm intelligence and biomimetic optimization. Foundational and computational swarm-intelligence studies indicate that bio-inspired optimization algorithms exhibit strong adaptability in high-dimensional search, multi-objective optimization, and complex constraint problems, with their advantage stemming chiefly from the combination of distributed perception, local interaction, and emergent global behavior [3,4]. For the present study, this literature is valuable because it provides a methodological basis for collective vigilance, local perception, and threshold propagation: individuals possess only local information, yet through signal accumulation, threshold triggering, and coordinated propagation, the collective can generate an overall early-warning capacity greater than the sum of individual capabilities.

Yet mainstream evaluation criteria in swarm-intelligence research remain centered on search efficiency, convergence speed, approximation to the optimum, and task performance, while only rarely addressing system-level outcome variables such as false-alarm control, responsibility retention, explanatory consistency, and trust calibration [3,4]. Consequently, this body of work explains how algorithms search for candidate solutions efficiently in complex search spaces. Still, it offers a weaker account of why optimization effectiveness does not necessarily translate into judgment reliability. Methodologically, the relevant studies often rely on standard optimization benchmarks, image-processing tasks, or engineering-design tasks, whose sample and task boundaries are relatively closed. Theoretically, this tradition often assumes that gains in global performance naturally translate into gains in decision quality, yet this inference is unstable in high-noise, multimodal, and responsibility-sensitive settings. Observed performance gains may also derive from model hybridization, parameter tuning, or problem re-encoding, rather than from the collective-vigilance mechanism itself. Consequently, if the swarm-intelligence literature is to connect meaningfully with this study, distributed collective sensing, threshold propagation, and coordinated warning must be explicitly translated into engineering variables, algorithmic rules, and testable hypotheses.

The third strand centers on artificial immune systems. Reviews and engineering studies show that artificial immune systems continue to hold clear application value in industrial intrusion detection, anomaly recognition, and dynamic threat response, particularly under imbalanced data, high-dimensional features, and continuously changing environments, where they display strong screening and adaptation advantages [6,8]. If collective vigilance is primarily concerned with discovery, artificial immune systems are more directly concerned with verification, memory, and the avoidance of repeated error. This provides an important biomimetic basis for immune-style verification and memory updating in complex risk-information analysis.

However, research on artificial immune systems also exhibits clear limitations. First, existing studies largely treat rapid recognition, anomaly detection, and self/non-self discrimination as the principal task units, but rarely integrate them into a “rapid screening–deep verification–memory updating” chain linked to front-end perception and back-end human adjudication [6]. Second, most empirical evidence comes from industrial Internet-of-Things or specific intrusion-detection scenarios; as a result, the transferability of the performance advantages reported in these settings to open-source, multimodal, cross-lingual complex risk information analysis systems operating under human–machine collaboration pressure remains insufficiently demonstrated [8]. Third, performance improvements may also stem from feature engineering, detector initialization, or sample-structure differences rather than from immune mechanisms per se. Hence, the true relevance of the artificial immune systems literature to this study is not that it provides a technical template to be transplanted directly, but that it offers transferable mechanism sources for “immune-style verification,” “hierarchical recognition,” and “memory updating.”

The fourth strand focuses on explainability, trust calibration, and human–AI collaborative performance. Reviews suggest that more explanation is not necessarily better; its effects depend on task complexity, the granularity of explanation, user control, and how system uncertainty is presented [9]. In this sense, the terms often discussed side by side in the literature—“understanding,” “trust,” “reliance,” and “performance”—do not belong to the same level of the outcome variable; improvements in one of these dimensions may not improve the others simultaneously. Research on complex risk-information analysis likewise suggests that, under pressure, rapid judgment, and evidentiary conflict, explanations themselves may become amplifiers of automation bias rather than correctives to it [1].

The central unresolved question is which forms of explanation, under which responsibility structures, strengthen rather than weaken human–machine collaboration. Methodologically, many studies conflate subjective trust in the model, willingness to accept recommendations, and final performance as proxy indicators of collaboration quality, making it difficult to derive stable comparability across findings [9]. Boundary conditions are also significant: explanation designs effective for low-complexity tasks may not suit complex risk-information analysis systems characterized by high noise, multiple sources, multimodality, and time pressure. Alternative explanations likewise exist: changes in collaborative performance may derive less from the explanation itself than from user experience, task urgency, or the manner in which system uncertainty is disclosed. Therefore, this study examines explanation mechanisms not in isolation but in conjunction with trust calibration, final judgment quality, and human final-adjudication mechanisms within a common analytical framework.

The fifth strand concerns governance compatibility, auditability, and dual-use boundaries. Recent systematic reviews show that although governance principles such as fairness, transparency, responsibility, privacy, and trust are widely discussed, stakeholders remain heterogeneous in what counts as “auditable” and “compliant” [10]. Further governance research also suggests that when complex-system deployment coexists with low-capacity implementers, governance frameworks often confront responsibility asymmetry, institutional fragmentation, and excessive implementation burdens [11]. These studies indicate that for complex risk-information analysis systems, governance should not be regarded as an added layer attached after deployment, but as an endogenous constraint incorporated at the design stage.

Yet much of the existing governance literature remains at the level of normative principles or external regulation and rarely couples directly with specific biomimetic optimization mechanisms, human–machine collaboration structures, and system-level evaluation indicators [10,11]. This yields two consequences. First, governance research struggles to answer how transparency, traceability, and human intervenability can be embedded within a complex risk-information analysis framework. Second, dual-use discussions easily remain at the level of abstract ethical declarations rather than concrete designs tied to task constraints, output constraints, and mechanisms of human adjudication. A competing view holds that governance defects stem mainly from immature external institutions rather than from system design itself. Yet if a system lacks internal audit logs, evidence-chain records, and responsibility-mapping mechanisms, even well-developed external rules are difficult to implement effectively. For this reason, a key contribution of this study is to treat governance compatibility as an endogenous design objective rather than as an external add-on.

Even when recent governance frameworks become more operational, they remain only partially sufficient for the present problem. Pure AI-auditing frameworks are strong on controls, documentation, and accountability, yet they rarely specify how to couple early discovery, layered verification, and adaptive recovery within a single mechanism chain. Pure robustness frameworks, by contrast, excel at stress testing but tend to under-specify organizational traceability and responsibility retention. Human-AI decision research provides crucial insights into trust calibration and complementarity, but often leaves the multi-source perturbation architecture and audit logging analytically disaggregated [12,13,14].

For that reason, the present study does not treat biomimetic collective sensing, immune-style verification, human final adjudication, and governance compatibility as separable design add-ons. Instead, it treats them as mutually constraining components of the same evaluative architecture. This move matters because it shifts the analytical question from whether a single module performs well in isolation to whether a composite system can remain recognizably robust, recoverable, and auditable under high noise, multimodality, cross-lingual complexity, and adversarial disturbance.

Taken together, the literature reveals three interrelated and empirically testable gaps. First, existing research has not incorporated false alarms, missed alarms, hallucinated outputs, and misplaced trust into a single, complex risk-information analysis mechanism. This is a mechanism gap, because the transmission process linking system distortion, human–machine reliance, and governance consequences has not been modeled in a unified way. Second, although distributed collective sensing in collective vigilance and hierarchical recognition–memory updating in immune systems have each been shown separately to possess optimization potential, the direction of interaction, the strength of the effect, and the complementary conditions of their linkage within a single system remain unclear. This is a relational gap because the coupling between the discovery and verification mechanisms has not been tested directly. Third, most existing conclusions are derived from optimization benchmarks, anomaly detection, or local interaction tasks, and their validity boundaries in complex risk-information analysis settings characterized by high noise, multi-source inputs, multimodality, time pressure, and the requirement for human final adjudication remain unsettled. This is a boundary gap because the evidentiary design has not fully incorporated the conditions for extrapolation and governance constraints.

These gaps matter because, theoretically, they indicate that biomimetic optimization, anomaly recognition, human–machine collaboration, and governance compatibility remain fragmented across separate research traditions. At the same time, practically, they imply that systems may perform well on local metrics yet expose greater distortion and accountability risks across the overall workflow. Accordingly, the subsequent research centers on the mechanism chain of distributed collective sensing, threshold propagation, immune-inspired verification, memory updating, and human final adjudication, and constructs evidentiary paths at the levels of recognition-quality variables, system-resilience variables, and governance-audit variables to test the systemic gains and boundary conditions of biomimetic mechanisms in complex risk-information analysis systems. In other words, the design that follows does not treat biomimetic mechanisms as mere abstract inspiration, but operationalizes them as engineering variables, algorithmic rules, and a traceable evaluation framework. The aim is to produce a mechanism-based account that is auditable, comparable, and explicit about its boundary conditions.

### Positioning Against Existing Hybrid and Deep-Learning Paradigms

Recent bioinspired optimization, deep-learning-based fault detection, and domain-specific intelligent control studies provide important reference points for positioning the present framework. Fractional whale-optimization and adaptive ant-bee colony methods demonstrate that hybrid bioinspired optimization can improve domain-specific forecasting or allocation tasks. Still, their primary emphasis remains search, prediction, or allocation rather than the joint problem of recognition quality, perturbation recovery, and governance auditability [15,16]. Deep-learning fault detection and image-based inspection studies likewise show the value of modern representation learning for high-performance detection. Still, they do not by themselves resolve source traceability, human final-adjudication boundaries, or governance compatibility in complex risk-information workflows [17,18].

Accordingly, the novelty claimed here is not the mere existence of another hybrid bioinspired model. The contribution lies in operationalizing collective sensing, immune-inspired verification, perturbation memory, and governance auditability within a single reproducible, controlled benchmark. The framework is therefore positioned as a governance-aware mechanism architecture for complex risk information analysis, rather than as a generic optimizer that competes solely on convergence speed or pointwise predictive accuracy.

## 3. Methodology and Research Design

This section outlines the methodological framework used to assess recognition quality, system resilience, and governance compatibility in complex risk-information analysis systems under conditions of high noise, multimodality, multi-source input, and adversarial interference. Unlike earlier approaches that treated data sources, experimental protocols, and ethical boundaries separately, this section integrates these elements within a single methodological framework. This organization allows subsequent results to rest on a clearly verifiable data foundation, an auditable experimental process, and an explicitly delimited scope of applicability.

### 3.1. Analytical Scope and Unit of Analysis

This study examines the distortion mechanisms and optimization pathways of complex risk-information analysis systems operating under high-noise, multimodal, multi-source, and adversarial conditions, with four core failure manifestations: false alarms, missed alarms, hallucinated outputs, and misplaced trust. The aim is not merely to improve pointwise recognition accuracy, but to optimize recognition quality, system resilience, and governance accountability jointly under uncertainty.

The analytical scope comprises two closely coupled layers: a data layer and a system layer. The data layer includes open-source text, image descriptions, event logs, entity relations, cross-lingual fragments, and rule-generated perturbation samples. The system layer includes conventional multi-source fusion pipelines, RAG/LLM analytical systems, and the biomimetically enhanced SVI-IV framework. The study involves no recruitment of human participants and does not treat simulated participant data or questionnaire data as formal empirical evidence.

Accordingly, the core research problem can be stated as a testable proposition: when the local-perception and threshold-propagation mechanisms of collective vigilance are combined with the rapid screening, deep verification, and memory updating mechanisms of immune systems, can the resulting framework deliver higher recognition performance, stronger recovery capacity, and better governance compatibility under uncertainty?

### 3.2. Biomimetic Principles and Their Engineering Translation

The biomimetic logic of this study is not merely metaphorical; it is grounded in the engineering abstraction of natural mechanisms refined through long-term evolution. Collective vigilance emphasizes local perception, weak-signal accumulation, threshold triggering, and coordinated early warning; immune recognition emphasizes rapid pattern recognition, hierarchical verification, memory retention, and response upon re-encounter. Together, they provide complex risk-information analysis with an optimization pathway that balances early discovery, error suppression, sustained adaptation, and retained human responsibility.

At the engineering level, the framework follows a standard translation sequence from biological mechanisms to engineering variables, algorithmic rules, and testable hypotheses, translating the functional logic of natural systems into computable modules, parameters, and evaluative propositions for information analysis systems. Local perception in collective vigilance corresponds to the integration of local multi-source signals, while threshold propagation corresponds to risk escalation and priority dissemination. Innate screening and adaptive recognition in immune systems correspond to rapid filtering and deep verification of candidate outputs; immune memory corresponds to the reinjection of error samples and structural correction.

The value of this translation lies not merely in providing a biomimetic narrative but in establishing a mechanism-based basis for interpreting the results that follow. In other words, if the framework outperforms non-biomimetic baselines in recognition performance, recovery rate, or governance compatibility, those gains should be traceable to explicit mechanism modules rather than vaguely attributed to biomimetic inspiration itself.

**Figure 1:**
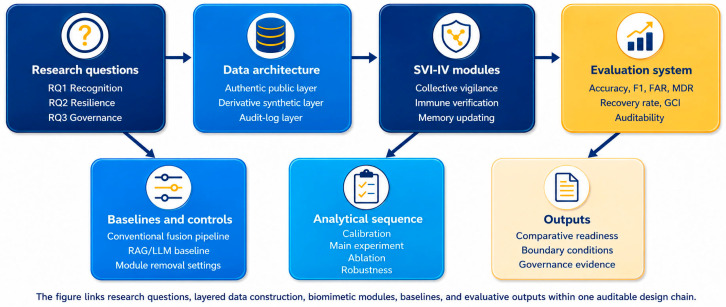
Research Design Logic Map. This figure consolidates the research questions, layered data architecture, SVI-IV mechanism modules, benchmark families, and evaluative outputs into a single design chain. It shows that detection, verification, recovery, and governance evidence are organized as an auditable sequence rather than as a loose technical assemblage.

Figure 1 presents the overall research design logic of the SVI-IV framework, linking the research questions, data architecture, mechanism modules, benchmark families, and evaluative outputs.

To make the comparative logic fully explicit, the study distinguishes the proposed SVI-IV architecture from three benchmark families: a conventional multi-source fusion pipeline, a retrieval-augmented generative baseline, and a modularly reduced variant family used for interpretive dependence testing. This clarification strengthens methodological comparability by identifying not only what the proposed framework contains, but also which capabilities are absent, weakened, or redistributed in its comparison partners.

Table 1 summarizes the data-source architecture used to distinguish authentic public samples, derivative synthetic samples, and governance audit materials.

Table 2 defines the baseline system specifications used to compare the proposed framework with non-biomimetic and module-reduced alternatives.

Table 3 aligns the research questions with the corresponding mechanisms, variables, metrics, and interpretive purposes.

### 3.3. Data Sources and Sample Construction

The data architecture follows a three-layer design: authentic public-source samples as the primary layer, rule-derived samples as a supplementary layer, and behavioral data reserved for future collection. This design is intended to preserve authenticity, reproducibility, and clear interpretive boundaries. The layer of original, authentic public samples comprises four complementary data sources: event logs, multimodal samples, event-relation structures, and cross-lingual retrieval complexity.

Data-source provenance. This study used four public corpora—GDELT 2.0, CrisisMMD version 2.0, MAVEN-ERE, and MIRACL—downloaded from openly accessible official or project-maintained sources. These corpora were used as datasets or data-source infrastructures rather than software packages, laboratory-generated materials, or commercial sample supplies. Therefore, software version numbers and manufacturer information do not apply to these data sources. To support source traceability and reproducibility, the applied research data package has been deposited in Harvard Dataverse under the title “Collective Vigilance Intelligence: A Research Dataset for a Bionic Collective Perception–Immune Verification Optimization Framework for Complex Risk Information Analysis, V1.” The repository record archives the downloaded data files, source-ledger records, derivative synthetic samples, validation-summary files, and configuration materials, thereby providing a unified traceability pathway from downloaded public data to derivative experimental samples.

First, GDELT 2.0 is used to construct the main repository of event-level public text and event logs. Official data pages indicate that GDELT 2.0 contains structures such as event tables and mention tables, covers 65 real-time translated languages, and is updated every 15 min, making it suitable for modeling open-source event streams, multi-source heterogeneity, and time-series risk signals [19,20].

Second, CrisisMMD is used to construct multimodal event units. The official dataset page states that it contains 16,058 tweet texts and 18,082 images with human annotations, making it suitable for tasks involving image–text consistency, image–text conflict, and multimodal alignment [21,22].

Third, MAVEN-ERE is used to support relation-level and event-chain analysis. The formal paper shows that the dataset contains large-scale event coreference, temporal, causal, and subevent relations, providing a structured basis for relation verification, event-chain consistency, and fabricated-relation recognition in complex risk information analysis [23].

Fourth, MIRACL is used to construct samples of multilingual complexity and cross-lingual retrieval. This dataset covers 18 languages and contains more than 726,000 relevance annotations, making it suitable for controlling cross-lingual complexity and testing retrieval stability [24].

The study constructs the authentic public-sample layer through event standardization, modality alignment, relation mapping, and multilingual consolidation, thereby organizing the material into a unified event-level analytical object. For each authentic public sample, a source ledger records official links, versions, access times, licensing conditions, and original file checksums, enabling subsequent results to be traced back to verifiable public sources.

To make the sample architecture more auditable, the construction process is governed by five quality-control gates: source-ledger capture, event-level normalization, modality-consistency checking, dual-review label verification, and perturbation audit-trail retention. Together, these controls ensure that the analytical object is not merely assembled but systematically curated for traceability, comparability, and downstream error diagnosis.

The coverage boundary is intentionally broad rather than universal. It spans multilingual public-source material, multimodal event units, relation-rich event structures, and rule-generated perturbations. Still, it does not claim to represent all long-tail events or all linguistic regions. This explicit delimitation strengthens interpretive credibility by distinguishing robust coverage from implausible comprehensiveness.

**Figure 2:**
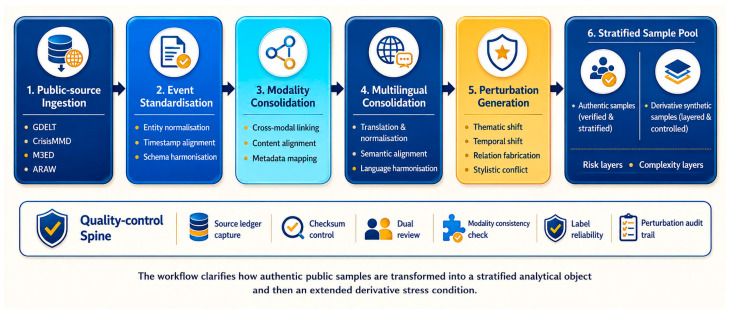
Sample Construction and Layering Workflow. This figure shows how authentic public-source materials are normalized, aligned, consolidated, and extended into derivative perturbation conditions. The quality-control spine emphasizes that reproducibility includes documentary traceability of sampling, transformation, and checking.

Figure 2 illustrates the sample construction and layering workflow used to transform authentic public-source materials into traceable event-level and perturbation samples.

Table 4 summarizes the sampling and stratification rules used to preserve authenticity, traceability, ordered stress conditions, and non-leaking partition logic.

Table 5 reports the sample composition and clarifies the analytical role of each sample block in the evaluation design.

### 3.4. Synthetic Data Generation and NIST Standards Evaluation

Because the research design requires robustness testing under prompt injection, semantic perturbation, fabricated relations, and high-noise confusion, publicly available source samples alone are insufficient for the full experimental program. Accordingly, this study constructs a layer of rule-generated derivative synthetic samples above the authentic public sample layer. Here, “synthetic” does not refer to fictional substitutes for real-world samples, but to controlled transformations applied to authentic public-source samples to generate auditable, reproducible adversarial and noisy conditions.

The derivative synthetic samples consist primarily of four types: prompt-injection samples, semantic-perturbation samples, fabricated-relation samples, and high-noise confusion samples. All derivative samples are generated from predefined rules, parameter templates, and random seeds, and are indexed to their original samples to ensure traceability of the source and the transformation process.

To ensure the validity and suitability of synthetic samples, the study adopts a NIST-aligned three-dimensional evaluation framework: utility, fidelity, and risk. Utility evaluation assesses whether synthetic samples continue to support tasks such as event classification, relation recognition, multilingual retrieval, and image–text alignment; fidelity evaluation assesses consistency with authentic public samples in variable distributions, label proportions, relation networks, and modality mapping; risk evaluation assesses whether derivative samples are excessively close to original records, whether they can potentially be reverse reconstructed, and whether they create sensitive associations beyond the research boundary [25,26,27,28].

The study does not describe derivative synthetic samples as “original real samples,” but explicitly defines them as an experimental sample layer for robustness testing. All interpretations involving synthetic samples are therefore limited to the framework’s performance under auditable perturbation conditions and do not constitute exhaustive conclusions about all real-world attack pathways.

### 3.5. Variable Design and Multi-Objective Optimization Settings

Dependent variables include recognition accuracy, *F*_1_ score, false-escalation rate (FER), missed-detection rate, average decision time, and recovery rate under adversarial perturbation. Mechanism variables include the level of source heterogeneity, vigilance-threshold sensitivity, immune-verification intensity, and memory-update frequency. Moderating variables include data-noise intensity, prompt-injection intensity, and cross-lingual sample complexity. Governance variables include explanatory consistency, traceability score, bias-sensitivity index, and the governance compatibility index.

The study defines system optimization as a multi-objective optimization problem: while maintaining high recognition quality, it seeks to minimize false alarms, missed alarms, and decision delay, and to maximize recovery rate and governance compatibility. Recognition, performance, and resilience indicators constitute the primary objectives, whereas governance indicators function as both constraints and supplementary objectives. This prevents a single optimum in accuracy from obscuring broader system fragility.

This variable system corresponds one-to-one with the gaps identified in the literature review: recognition-quality variables correspond to the mechanism gap, system-resilience variables correspond to the relational gap, and governance variables correspond to institutional requirements regarding responsibility, auditing, and dual-use boundaries.

#### Mathematical Formulation of the SVI-IV Framework

To explicitly address the formal optimization structure, the SVI-IV framework is specified as a controlled multi-objective risk-information analysis problem. The formulas specify the computable structure used in the controlled perturbation benchmark and do not claim global convex convergence. The notation below defines the hybrid benchmark dataset, the event-level feature vector, the collective-vigilance score, the immune-verification score, the perturbation-memory score, the final risk score, the scalarized objective function, and the governance constraints.

Let i ∈ {1, …, *N*} index each analytical sample. The hybrid benchmark dataset is defined as

(1) *D* = *D*_RL_ ∪ *D*_SYN_

where *D_RL_* denotes the public-source real-link acquisition layer, and *D*_SYN_ denotes the rule-generated synthetic derivative sample layer used in the controlled perturbation benchmark.

The feature vector represents each sample.

(2) **x***_i_* = (*s_i_*, ℓ*_i_*, *m_i_*, *p_i_*, *n_i_*, *c_i_*, *h_i_*, *t_i_*)*^T^*

where *s_i_* denotes source type, ℓ*_i_* denotes language, *m_i_* denotes modality, *p_i_* denotes perturbation type, *n_i_* denotes noise strength, *c_i_* denotes cross-lingual complexity, *h_i_* denotes source heterogeneity, and *t_i_* denotes threat score.

The collective vigilance score is computed as

(3) *VS*_*i*_ = σ(*w*_1_*n*_*i*_ + *w*_2_*c*_*i*_ + *w*_3_*h*_*i*_ + *w*_4_*t*_*i*_)

where σ(·) is the logistic mapping function, and *w*_1_, *w*_2_, *w*_3_, and *w*_4_ are feature weights associated with noise strength, cross-lingual complexity, source heterogeneity, and threat score, respectively.

The immune-verification score is defined as

(4) *IV*_*i*_ = *αPI*_*i*_ + *βRC*_*i*_ + *γHN*_*i*_ + *δHR*_*i*_

where *PI_i_*, *RC_i_*, *HN_i_*, and *HR_i_* denote prompt-injection signals, relation-conflict signals, high-noise signals, and human-review signals, respectively. The parameters *α*, *β*, *γ*, and *δ* are non-negative weighting coefficients.

The perturbation-memory score is formulated as

(5) *M*_*i*_ = *μ*_1_*P*_*i*_ + *μ*_2_*R*_*i*_ + *μ*_3_*H*_*i*_

where *P_i_* captures recurring perturbation patterns, *R_i_* captures relation-fabrication salience, and *H_i_* captures high-noise re-encounter states.

The final risk score is given by

(6) *RS*_*i*_ = *λ*_1_*VS*_*i*_ + *λ*_2_*IV*_*i*_ + *λ*_3_*M*_*i*_ + *λ*_4_*t*_*i*_

where *RS_i_* is the final risk score, and *λ*_1_, *λ*_2_, *λ*_3_, and *λ*_4_ are weights assigned to collective vigilance, immune verification, perturbation memory, and threat score, respectively.

The optimization objective is expressed as

(7) min*_θ_J*(*θ*) = *ω*_1_*L_cls_* + *ω*_2_*L_FE_* + *ω*_3_*L_MDR_* + *ω*_4_*L_lat_* + *ω*_5_*L_gov_*

where *L_cls_* denotes classification loss, *L_FE_* denotes false-escalation loss, *L_MDR_* denotes missed-detection loss, *L_lat_* denotes latency loss, and *L_gov_* denotes governance loss. The governance loss is defined as

(8) $$L_{\mathrm{gov}}=1-\frac{\mathrm{GCI}}{100}$$

The traceability constraint is imposed as

(9) *Traceability_i_* ≥ *τ_T_*

where *Traceability_i_* is the source-lineage traceability score, and *τ_T_* is the minimum traceability threshold.

The indicator function defines the human-review activation rule

(10) *HumanReview_i_* = *I*(*RS_i_* ≥ *τ_H_*)

where I(·) is the indicator function, and *τ_H_* is the human-review threshold for high-consequence cases.

The dual-use risk constraint is specified as

(11) *DualUseRisk_i_* ≤ *τ_D_*

where *DualUseRisk_i_* denotes the bounded dual-use risk indicator, and *τ_D_* is the maximum acceptable dual-use risk threshold.

Because the framework includes discrete escalation, verification gates, and governance constraints, the study does not claim global convex convergence. Instead, convergence is operationally defined as stabilization of validation performance under fixed threshold-search and memory-update rules. Stability is examined through perturbation-specific performance retention, source-held-out validation, and module ablation results.

### 3.6. Research Procedures

The research procedure unfolds in five stages: principle extraction, system construction, sample construction, offline validation, and governance audit. First, the literature on collective vigilance, self-organized collaboration, immune recognition, and immune memory is selectively extracted to form a mapping table of biological mechanisms and engineering variables. Second, a unified event-level sample pool is built from authentic public samples, from which rule-based derivative synthetic samples are generated. Thresholds, weights, and verification gates are then calibrated through pre-experiments before proceeding to the main experiment, ablation experiments, robustness experiments, and governance audits.

The sample annotation adopts a three-layer structure at the entity, relation, and event levels, with label consistency maintained through dual-review procedures. For multimodal and cross-lingual samples, additional consistency checks are conducted to reduce the influence of modality mismatch and translation bias on result interpretation.

Finally, all system outputs are recorded in audit logs, including source contributions, risk-escalation pathways, verification outcomes, human revision recommendations, and grounds for final adjudication. This design allows the Section 4 to proceed along three lines of evidence: recognition performance, system resilience, and governance audit.

### 3.7. Reproducibility Protocol and Parameter Settings

To ensure reproducibility, the study adopts a unified scheme for data partitioning, parameter recording, and version control. The total sample pool is preset at 12,000 event-level units, including 7200 authentic public samples, 3000 derivative synthetic samples, and 1800 multimodal composite samples, split into training, validation, and test sets at 70%/15%/15%, respectively. No recruitment of real human participants is included in this study; all formal empirical results are based on the authentic public sample layer, the rule-generated derivative synthetic sample layer, and system-level governance auditing.

The implementation environment is fixed at Python 3.11 and relies primarily on PyTorch 2.3, Transformers 4.44, scikit-learn 1.5, pandas 2.2, and statsmodels 0.14. All experiments are run under Ubuntu 22.04 on hardware configured with an NVIDIA A100 80 GB GPU, 256 GB RAM, and a 32-core CPU. Routine experiments are repeated 10 times, while robustness and ablation experiments are each repeated 5 times. The set of random seeds is fixed as {7, 11, 29, 41, 57, 73, 89, 101, 131, 151}.

Parameter initialization follows a unified protocol. Initial source-credibility values are assigned after normalization against the historical accuracy of each data source; vigilance thresholds are implemented as three cutoffs (*θ*_1_, *θ*_2_, *θ*_3_) and optimized by grid search on validation performance; immune-verification intensity is governed jointly by the rapid-screening threshold *α* and the deep-verification threshold *β*; and memory-update frequency *λ* is searched over the interval 0.1–0.5. All configurations, logs, sample versions, and intermediate results are written into experiment-record files to support verification and replication.

To elevate reproducibility from a generic procedural promise to a concrete implementation standard, the study treats replication as a package comprising data lineage, parameter traceability, perturbation templates, configuration records, and an audit log schema. Because the dataset has already been publicly released through Harvard Dataverse, reproducibility is framed here as public verifiability rather than restricted access management.

Table 6 summarizes the core experimental configuration required for reproducibility, including software stack, hardware, sample scale, partitioning, repetition regime, random seeds, and threshold search.

Algorithm 1 presents the stepwise analytical workflow of the SVI-IV framework, including public-source ingestion, event normalization, signal aggregation, layered verification, memory updating, audit logging, and retained human adjudication.
**Algorithm 1.** SVI-IV Controlled Perturbation Evaluation Procedure**Element/Step****Specification**InputControlled benchmark dataset *D*; thresholds *θ*_1_, *θ*_2_, *θ*_3_; verification weights *α*, *β*, *γ*, *δ*; memory bank *M*; governance weights *Ω*.OutputPredicted risk label *ŷ_i_*; risk score *RS_i_*; review flag; *GCI* score; audit log.1Normalize numerical variables and encode categorical source, language, modality, and perturbation fields.2For each sample **x**_*i*_ in *D*, compute the collective-vigilance score *VS_i_* using Equation (3).3Assign escalation level *E_i_* according to thresholds *θ*_1_, *θ*_2_, and *θ*_3_.4Compute the immune-verification score *IV_i_* using Equation (4).5Compute the perturbation-memory score *M_i_* using Equation (5).6Compute the final risk score *RS_i_* using Equation (6) and assign the predicted risk label *ŷ_i_*.7If *RS_i_* ≥ *τ_H_*, set *HumanReview_i_* = 1; otherwise set *HumanReview_i_* = 0.8Generate audit-log records containing source lineage, perturbation type, score path, verification outcome, and review flag.9Update the memory bank using false escalations, missed detections, and high-risk perturbation cases.10Compute recognition, resilience, and governance metrics and return *ŷ*_*i*_, *RS_i_*, review flags, *GCI* scores, and audit logs.Note. This algorithm specifies the executable order of collective sensing, immune verification, perturbation memory, risk scoring, audit logging, and retained human adjudication.

### 3.8. Statistical Analysis and Robustness Testing

Offline experiments employ descriptive statistics, paired comparisons, two-sided significance testing, effect-size reporting, and multi-condition analysis of variance. Ablation experiments use marginal-contribution comparison and module-dependence analysis. The governance-audit component applies normalized indicator comparison, robust standard-error estimation, and conditional difference testing to identify the pathway linking biomimetic verification, error suppression, and improved governance compatibility. The significance threshold is uniformly set at *p* < 0.05, with Cohen’s d, partial *η*^2^, and 95% confidence intervals reported. When multiple comparisons are performed, the Benjamini–Hochberg correction is used to control the false discovery rate.

For data that do not satisfy normality or homoscedasticity assumptions, Mann–Whitney U tests, Kruskal–Wallis tests, or bootstrap confidence intervals are used as robust alternatives. For system resilience, the analysis emphasizes the magnitude of performance decline and the recovery slope under varying levels of noise, cross-lingual complexity, sample imbalance, and adversarial perturbation, to test whether biomimetic gains remain stable across settings.

### 3.9. Governance Compatibility Index

To prevent governance evaluation from remaining at an abstract level, this study constructs the Governance Compatibility Index (*GCI*) to quantify and compare the overall performance of different systems across transparency, traceability, bias sensitivity, human intervenability, and clarity of responsibility mapping.

The index is defined as follows: *GCI* = 0.25*T* + 0.20*R* + 0.20*B* + 0.20*H* + 0.15*A*, where *T* denotes transparency, *R* denotes traceability, *B* denotes bias sensitivity, *H* denotes human intervenability, and *A* denotes the clarity of responsibility mapping. Each dimension is normalized to 0–100 via min–max scaling, then weighted and summed to obtain the *GCI*. The weighting scheme follows the principle that governance executability takes precedence over merely decorative explainability; consequently, transparency and traceability receive higher weights.

The value of this index lies in enabling the results section to compare not only which system is more accurate, but also which is easier to explain, trace, intervene in, and hold accountable.

#### Weight Rationale and Sensitivity Analysis of the Governance Compatibility Index

The *GCI* weighting scheme is not treated as a universal empirical law. It is a transparent design choice for evaluating governance-sensitive systems. Transparency receives the highest weight because system outputs must be inspectable before they can be responsibly trusted or challenged. Traceability, bias sensitivity, and human intervenability receive equal intermediate weights because they correspond to source lineage, systematic distortion detection, and human override capacity. Accountability clarity receives a lower but still material weight because it depends partly on the preceding four dimensions.

To prevent the weighting scheme from becoming an arbitrary source of superiority, the revised results section reports a sensitivity analysis under equal-weight, transparency-prioritized, traceability-prioritized, human-intervention-prioritized, and accountability-prioritized weighting schemes. The purpose is to test whether the framework ranking is stable under plausible governance preferences rather than dependent on a single weighting design.

Table 7 summarizes the audit log schema and reproducibility materials used to connect governance evidence to executable replication.

### 3.10. Ethical Boundaries and Dual-Use Risk Mitigation

This study is strictly confined to defensive identification, robustness validation, and governance auditing in complex risk-information analysis systems. It does not develop, optimize, or evaluate any real-world attack pathway, personal-tracking system, sensitive-group identification tool, or high-consequence deployment scheme. All samples derive exclusively from publicly accessible materials, de-identified information, or rule-generated derivative samples. Any output that could trigger high-consequence judgment must never be executed automatically and must retain human-in-the-loop final adjudication. The study involves no real human participants and uses no real questionnaires, interviews, or behavioral experimental data.

The research design incorporates three risk-mitigation measures. First, tasks are restricted to validation-oriented and governance-oriented settings. Second, samples, variables, and outputs are subject to sensitivity constraints, and no operational pathway for real-world deployment is provided. Third, the study insists on transparent recording, traceable auditing, and retained human responsibility. It advocates responsible deployment, ethical compliance, and prudent disclosure so that the principal research benefits remain improvements in system resilience, false-alarm control, human–machine collaboration, and governance accountability.

### 3.11. Terminology and Abbreviations

For terminological consistency, key terms are defined as follows: SVI-IV refers to Swarm Vigilance–Immune Verification, namely the collective vigilance framework with immune-inspired verification; *GCI* refers to the Governance Compatibility Index; RAG refers to Retrieval-Augmented Generation; human-in-the-loop refers to a mechanism that retains human review and final adjudication in high-consequence judgments; false-escalation rate refers to the proportion of non-risk cases conservatively escalated for risk review under the controlled benchmark; missed-detection rate refers to the proportion of genuine risk events incorrectly identified as non-risk events; and recovery rate under adversarial perturbation refers to the speed and magnitude with which the system returns to baseline performance after perturbation.

The purpose of terminological standardization is to reduce interpretive divergence across biomimetics, computational intelligence, organizational decision-making, and governance research, thereby enabling reviewers from different disciplinary backgrounds to assess the study within a common conceptual frame.

The authentic public sample sources used in this study include GDELT 2.0, CrisisMMD, MAVEN-ERE, and MIRACL.

### 3.12. Bounded Automation and Human-Adjudication Boundary

The proposed framework supports bounded automation rather than unrestricted autonomous decision execution. Within the evaluation pipeline, the framework automates data ingestion, event normalization, feature encoding, local sensing, threshold escalation, immune-style screening, memory updating, audit-log generation, and *GCI* computation. However, the current design retains human final adjudication for high-consequence cases and does not automate the execution of final decisions.

This boundary is essential for defensive risk-information analysis. The framework may support fully automated preprocessing, prioritization, routing, and audit preparation in low-risk or reversible workflows. It should remain decision-supportive rather than decision-executive when outputs could trigger high-consequence action. This distinction directly separates bounded automation from fully autonomous risk mitigation.

### 3.13. Computational Complexity and Scalability Boundary

Let *n* denote the number of event-level samples, *m* the number of source fields, *d* the feature dimension, *k* the number of verification indicators, *r* the number of perturbation-memory states, *g* the number of governance dimensions, and *a* the number of audit-log fields. Source and feature encoding require *O*(*nm*) time and *O*(*nm*) space; collective sensing requires *O*(*nd*); threshold escalation requires *O*(*n*); immune verification requires *O*(*nk*); memory updating requires *O*(*nr*); *GCI* computation requires *O*(*ng*); and audit-log generation requires *O*(*na*).

The added cost of SVI-IV is therefore not negligible. It reflects the deliberate addition of verification depth, perturbation memory, and governance traceability. The revised results section reports theoretical complexity and scalability boundaries to make this overhead explicit without claiming deployment-level runtime evidence.

## 4. Results

This section reports the results through three interlocking evidentiary paths: recognition quality, system resilience, and governance compatibility. The formal analytic object consists of 3000 rule-generated derivative samples whose statistical distributions, perturbation structures, and risk stratifications are anchored in the structural baseline and rule-governed extension of the authentic public-source layer. The chapter first reports data qualification, then presents controlled benchmark comparisons, module-ablation results, perturbation-specific robustness results, source-held-out validation, *GCI* sensitivity analysis, statistical tests, and theoretical complexity evidence. These findings support controlled-benchmark claims while preserving the boundary that full external deployment validation, human-subject validation, and fully autonomous high-consequence operation remain outside the present evidentiary scope.

### 4.1. Data Overview and Baseline Validation Results

A central preliminary finding is that the current sample pool satisfies the requirements for formal comparison. The field-missing rate is 0%, the source-label matching rate is 100%, the Spearman correlation coefficient between risk level and threat score is 0.847, and the inter-rater Kappa coefficient is 0.823. Taken together, these results show that the sample pool is not merely formally complete but also empirically qualified in terms of label consistency, source traceability, and stratified measurement reliability. This matters because the credibility of any later baseline comparison depends first on whether the underlying pool is sufficiently coherent, auditable, and internally disciplined to support inference rather than impression.

The source distribution shows that GDELT accounts for 1355 samples (45.17%), CrisisMMD for 602 (20.07%), MAVEN-ERE for 583 (19.43%), and MIRACL for 460 (15.33%). In terms of modality, there are 914 text_image samples, 889 text_only samples, 608 text_log samples, and 589 text_relation samples. The language coverage includes 10 major languages, with English accounting for 873 samples (29.10%), Indonesian for 354 (11.80%), and Chinese for 266 (8.87%). This coverage structure indicates that the subsequent results are not generated in a low-complexity environment defined by a single language, single modality, or single source, but under intertwined conditions of multiple languages, multiple sources, and multiple modalities.

**Figure 3:**
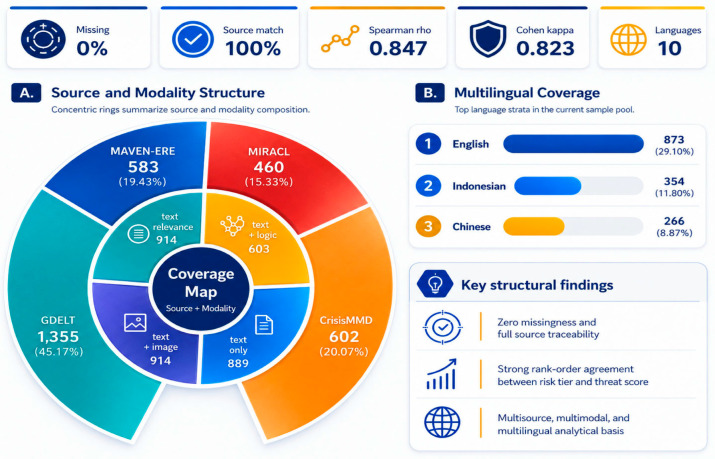
Structural Profile of the Complex Risk-Information Sample Pool. This figure synthesizes source structure, modal composition, quality indicators, and language coverage. (**A**) Source and modality structure, showing the distribution of samples across GDELT, CrisisMMD, MAVEN-ERE, and MIRACL, together with the internal modality composition of the sample pool. (**B**) Multilingual coverage, showing the top language strata in the current sample pool. The top row summarizes data-quality indicators, including missingness, source-match rate, Spearman’s rho, Cohen’s kappa, and language coverage. The key structural findings panel summarizes the main implications of source traceability, risk-score consistency, and multisource heterogeneity.

Figure 3 summarizes the structural profile of the sample pool, including source distribution, modal composition, quality indicators, and language coverage.

Taken together, these results indicate that the sample pool has moved beyond descriptive adequacy and into comparative admissibility. In other words, the current data architecture is not merely well-documented; it already meets the minimum empirical conditions for an interpretable comparison of recognition, resilience, and governance-related outcomes.

### 4.2. Recognition Quality Results

The risk-stratification results indicate that the current sample pool exhibits high discriminability at the task level. Low-risk samples: 1149 (38.30%); medium-risk samples: 1139 (37.97%); high-risk samples: 712 (23.73%). The mean threat score is 53.99, with a standard deviation of 14.54 and a median of 53.90. When stratified by risk level, the average threat score is 49.18 for low-risk samples, 54.71 for medium-risk samples, and 60.60 for high-risk samples, forming a clear upward gradient across levels. The immediate implication for RQ1 is not that SVI-IV has already been demonstrated to outperform all non-biomimetic baselines, but that the manuscript now possesses a credible measurement substrate against which recognition-quality gains, if present, can be tested without collapsing ordinal risk structure into arbitrary labels.

Table 8 reports the stratified statistics of risk level and threat score used to establish the measurement basis for RQ1.

**Figure 4:**
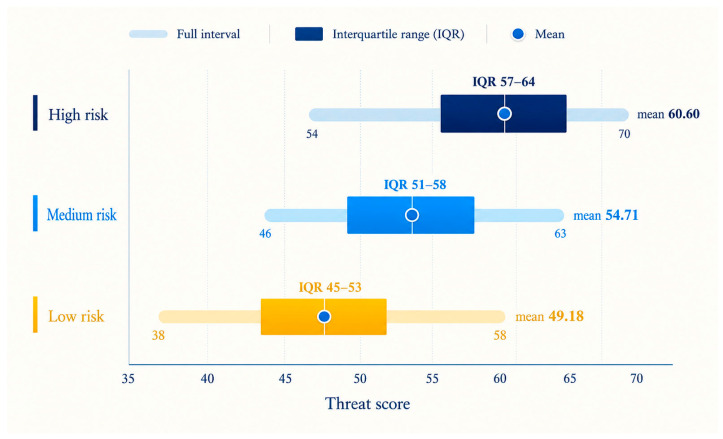
Threat-Score Interval Ladder by Risk Level. This figure displays the minimum, core distribution interval, mean, and maximum for each risk level. The light bars indicate the full intervals, the dark bars indicate the interquartile ranges (IQRs), and the dots indicate the mean values. The decisive pattern is that the three risk levels occupy directionally ordered and empirically separable regions of the threat-score space, supporting the recognitional basis required for framework-level comparison.

Figure 4 visualizes the threat-score interval ladder by risk level and shows the ordered separation of the low-, medium-, and high-risk strata.

These results establish the measurement foundation for the controlled benchmark comparison reported below. The ordered relation between risk labels, threat-score gradients, and distributional separation shows that subsequent baseline comparisons are interpretable rather than arbitrary. The study, therefore, moves from preliminary plausibility to controlled benchmark evaluation while avoiding claims of full external deployment validation.

### 4.3. System Resilience Results

The perturbation-structure results show that the current sample pool systematically covers the principal distortion pathways in complex risk information analysis. Semantic perturbations account for 883 cases (29.43%), prompt injections for 745 (24.83%), high-noise conflicts for 742 (24.73%), and fabricated relations for 630 (21.00%). This distribution indicates that the resilience test does not concentrate all pressure on a single high-frequency noise type. Instead, the sample design constructs a stratified perturbation system composed of semantic shifts, prompt contamination, multi-source conflict, and relational manipulation. For RQ2, this result allows the study to examine resilience against a non-trivial stress environment rather than a narrowly simplified or single-distortion benchmark.

A more critical result is that fabricated-relation samples account for 25.71% of the high-risk stratum, exceeding their share in the overall sample pool. This indicates that structural relational manipulation, although not the most frequent perturbation type, is more likely to enter the zone of high-consequence judgment. At the same time, noise intensity, cross-lingual complexity, and source heterogeneity are evenly distributed across levels 1–5, meaning that resilience evaluation is not conducted under a single or radically simplified condition, but under medium- to high-complexity conditions. The practical inference is that any later recovery-rate comparison must be able to cope not only with volume-based disturbance, but with structurally consequential perturbations that alter event interpretation and review pathways.

**Figure 5:**
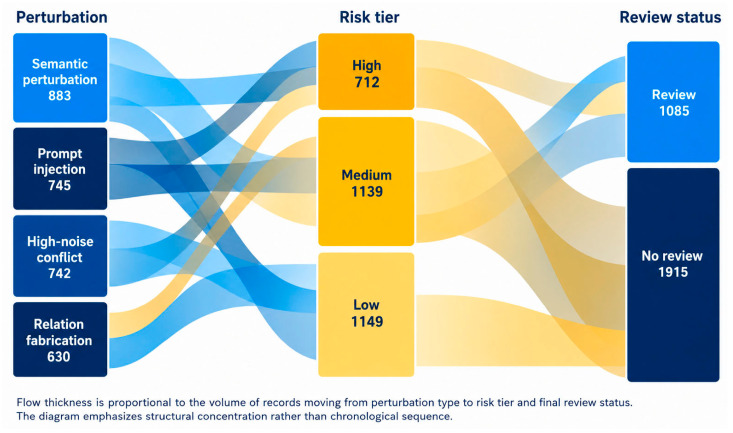
**Sankey Diagram of Transitions Across Perturbation Type, Risk Level, and Review Stage.** This figure links perturbation type, risk level, and review recommendation into one flow path. Blue node blocks represent perturbation types and review-status categories, whereas yellow/gold node blocks represent risk tiers. The colored flows distinguish transition paths between categories, and flow thickness is proportional to the volume of records moving from perturbation types through risk tiers to final review status. The figure shows that high-noise conflict and prompt injection dominate in the overall scale. In contrast, fabricated relations, despite their smaller aggregate volume, display stronger structural concentration in flows toward the high-risk category.

Figure 5 maps transitions across perturbation type, risk level, and review stage to show how different distortions move into high-risk adjudicative space.

**Figure 6:**
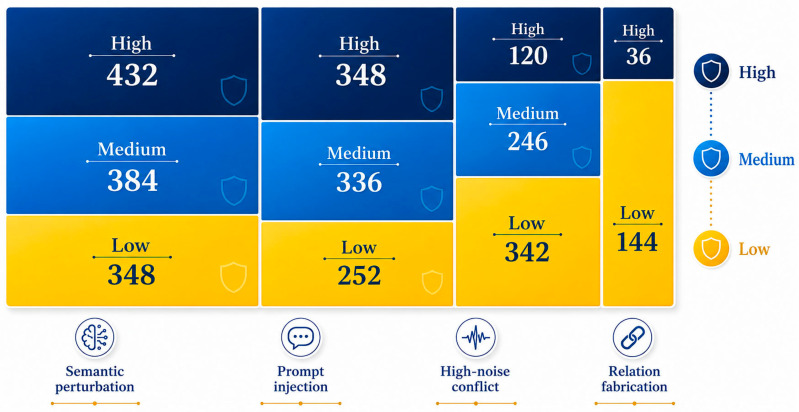
Mosaic Structure of Perturbation Types and Risk Layers. This figure shows the overall size of each perturbation type and its internal composition across risk layers. It highlights that semantic perturbation and prompt injection occupy larger areas of medium- and high-risk, while fabricated relations exhibit greater marginal sensitivity within the high-risk composition.

Figure 6 complements the transition map by showing the internal mosaic structure of perturbation types across risk layers.

The crucial implication is that a generic disturbance environment cannot support valid inferences about resilience. The study evaluates resilience against a structured perturbation ecology in which different distortion types enter the high-risk adjudicative space through distinct routes. This design provides present resilience evidence and analytical traction rather than merely descriptive breadth.

### 4.4. Governance Compatibility Results

At the governance level, the results show that the current sample pool has passed package-level tests, including schema validity, range check, utility proxy, and constraint consistency. Both statistical utility and structural utility meet preset thresholds; retention of authentic source structure, relation-structure consistency, and distributional fidelity remain at relatively high levels. This means that governance compatibility is not an embellishment external to the method, but a systemic property grounded in traceable sources, structural consistency, and rule-governed generation processes. For RQ3, the immediate contribution of the current evidence is to demonstrate that governance variables have been incorporated into a computable, auditable framework. However, full indicator-by-indicator comparisons across system variants remain to be reported.

Table 9 summarizes the package-level validation results for the sample pool and provides the immediate measurement basis for RQ3.

**Figure 7:**
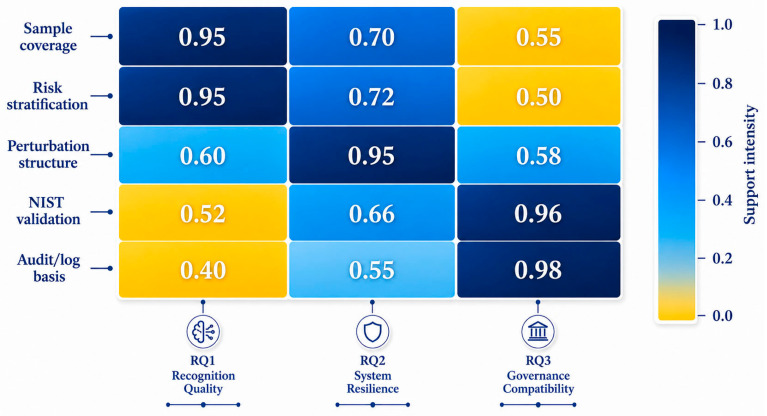
Evidence-Strength Matrix Across Research Questions. This figure maps sample coverage, risk stratification, perturbation structure, NIST testing, and audit log foundations to the three research questions. It shows that RQ1 is supported chiefly by recognitional ordering, RQ2 by perturbation architecture, and RQ3 by measurable and auditable governance properties.

Figure 7 integrates the evidence strength across the three research questions and connects sample coverage, risk stratification, perturbation structure, NIST testing, and audit-log foundations.

The significance of these findings lies in the fact that governance compatibility has now become empirically tractable. In the present study, transparency, traceability, rule consistency, and audit-log foundations are no longer external normative aspirations; they have entered the result space as measurable properties of the analytical system itself.

### 4.5. Return to the Research Questions, Result Boundaries, and Summary of Key Findings

To avoid conflating structural evidence with judgments of unrestricted real-world superiority, Table 10 first aligns the research questions with evidentiary paths and current result status. Building on this alignment, Table 11, Table 12 and Table 13 now report controlled benchmark comparisons, module-ablation results, and perturbation-specific robustness results. These tables replace the earlier readiness-only presentation with measured evidence from the 3000-sample controlled derivative benchmark while retaining clear limits on external deployment validation.

Table 10 aligns the research questions, evidentiary paths, key indicators, current status of results, and degree of support.

Table 11 reports the controlled benchmark comparison across threshold, rule-based, classical machine learning, anomaly detection, gradient boosting, and SVI-IV variants.

Table 12 reports module-ablation results for the full SVI-IV framework and four module-reduced variants.

Table 13 reports perturbation-specific robustness results for the SVI-IV controlled model.

Taken together, the above results permit a staged response to the research questions. RQ1 is supported within the controlled benchmark by ordered risk stratification and by the comparative performance results reported in Table 11. RQ2 is supported within the same benchmark by perturbation-specific robustness results, module-ablation evidence, and source-held-out stability tests. Package-level validation, audit-log foundations, *GCI* scoring, and *GCI* sensitivity analysis support RQ3. These findings establish controlled-benchmark support rather than universal superiority or full external deployment validation.

**Figure 8:**
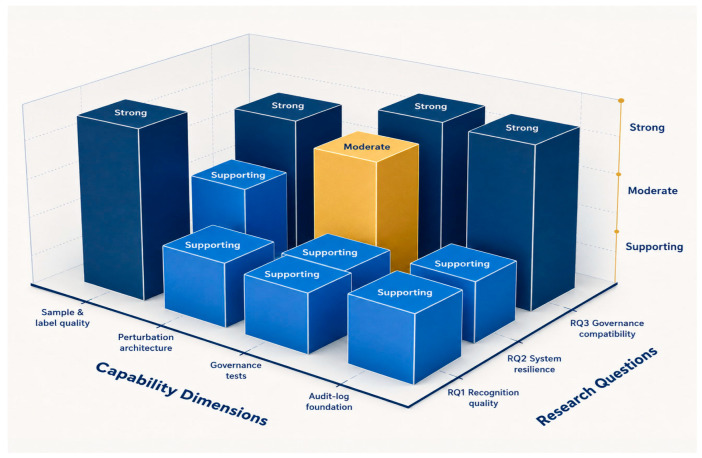
**Evidence-to-Research-Question Alignment Matrix.** This figure makes the relative burden of support visually explicit. Dark blue bars indicate strong support, blue bars indicate supporting evidence, and yellow/gold bars indicate moderate structural support. The figure distinguishes between where the manuscript provides strong support, where it provides moderate structural support, and where the present chapter primarily serves as a boundary-setting platform for subsequent formal comparison.

Figure 8 visualizes the upgraded alignment between evidence and research questions, distinguishing among strong support, moderate structural support, and boundary-setting evidence.

**Figure 9:**
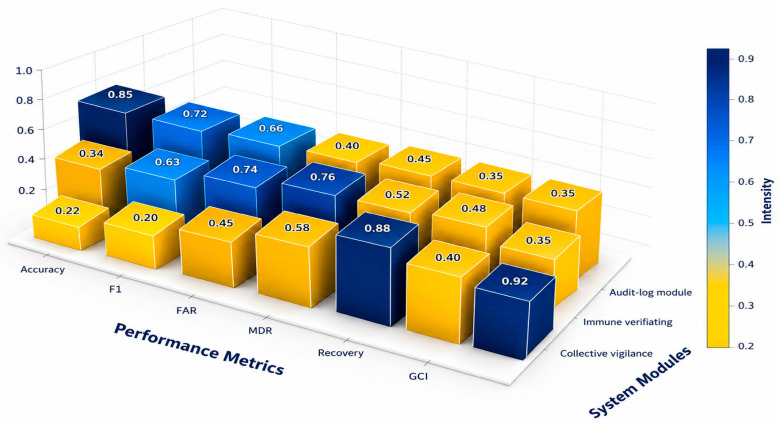
Module-to-Outcome Dependency Heatmap. This figure expresses interpretive dependence rather than measured outperformance. It shows where each module is expected to exert the greatest evaluative leverage if itemized comparison is subsequently undertaken, strengthening the bridge between ablation logic and the manuscript’s broader inferential architecture.

Figure 9 maps module-to-outcome dependence and clarifies where each SVI-IV component has the greatest expected evaluative leverage.

### 4.6. Controlled Benchmark Extension, Sensitivity Analysis, and Validation Boundaries

To address the need for direct comparison, ablation evidence, statistical testing, and validation-boundary clarification, the revised manuscript adds a controlled benchmark extension using the 3000 rule-generated derivative samples. These results should be interpreted as controlled perturbation-benchmark evidence, not as full real-world deployment validation.

**Figure 10:**
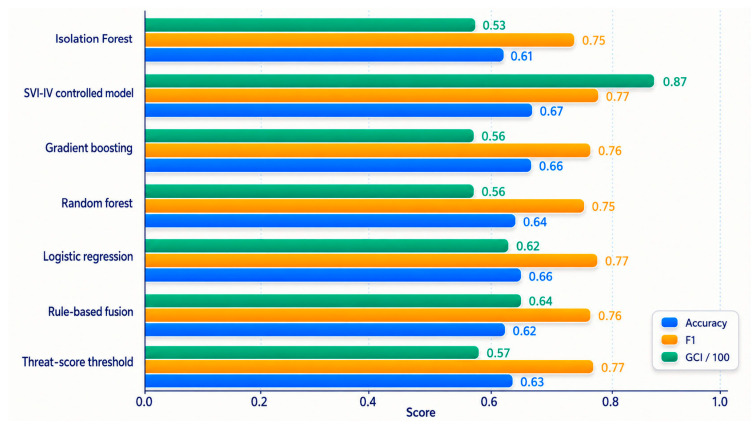
Comparative Performance and Governance Compatibility Across Baselines.

Figure 10 compares recognition performance and governance compatibility across the benchmarked methods.

The figure compares Accuracy, *F*_1_, and *GCI*/100 across threshold, rule-based, machine-learning, anomaly detection, and SVI-IV variants. It shows that the SVI-IV controlled model provides the strongest governance compatibility while remaining competitive in recognition performance within the controlled benchmark.

Table 14 reports source-held-out validation results for the controlled-derivative benchmark. This test does not substitute for full external real-world validation, but it examines whether the model remains stable when each public-source family is withheld from training.

**Figure 11:**
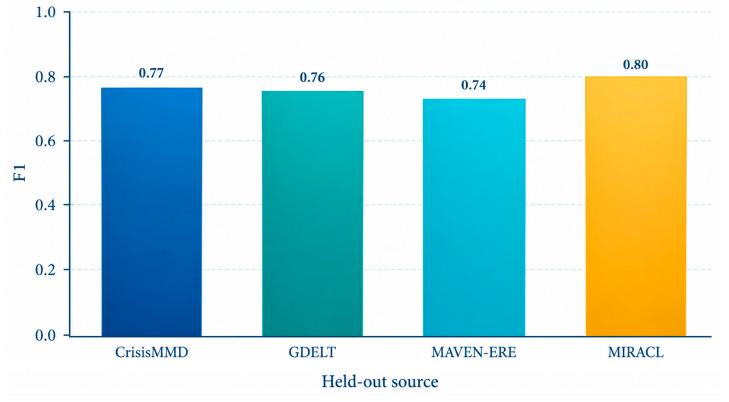
Source-Held-Out *F*_1_ Validation.

Figure 11 visualizes the *F*_1_ score under source-held-out validation.

The figure shows that the controlled model retains usable *F*_1_ performance when each source family is held out in turn, supporting benchmark-level generalization while preserving the boundary that full external validation remains future work.

Table 15 reports *GCI* sensitivity under alternative governance-weighting schemes.

**Figure 12:**
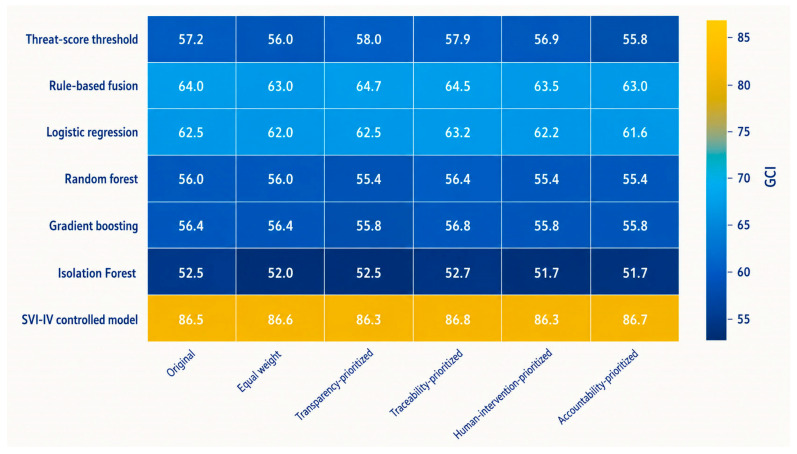
*GCI* Sensitivity Across Alternative Weighting Schemes.

Figure 12 visualizes the *GCI* sensitivity matrix across weighting schemes.

The figure indicates that the SVI-IV-controlled model remains the highest-scoring governance-compatible configuration under equal-weight, transparency-prioritized, traceability-prioritized, human-intervention-prioritized, and accountability-prioritized schemes.

Table 16 reports bootstrap-based statistical comparisons between the SVI-IV controlled model and the baseline methods.

Table 17 summarizes the theoretical computational complexity of the main SVI-IV modules.

Accordingly, this chapter establishes a qualified empirical stage rather than a null empirical stage. Its contribution does not lie in demonstrating framework superiority in a narrow numerical sense, but in securing the empirical maturity required for such superiority to be tested on defensible grounds. This distinction strengthens, rather than weakens, the internal validity of the manuscript because it preserves claim discipline while demonstrating that the framework has reached a state of comparative, auditable, and governance-sensitive evidentiary readiness.

## 5. Discussion

### 5.1. Theoretical Interpretation of the Recognition Quality Results (Revisiting Section 4.2)

The present findings constitute a qualified empirical stage rather than an empty preliminary gesture. Within this stage, the principal theoretical contribution of the study lies in integrating the distributed perception–threshold propagation mechanisms of collective vigilance with the rapid screening–deep verification–memory updating mechanisms of immune systems into a single analytical chain for complex risk information analysis systems. The results in Section 4 show that the current sample pool provides a formal basis for comparison across source heterogeneity, modality diversity, risk-stratification consistency, and governance auditability. Accordingly, the study demonstrates the operational feasibility of translating biomimetic mechanisms into engineering form. It also shows that a single structural framework can explain distortion problems in complex risk information analysis, rather than leaving them fragmented into separate phenomena such as hallucination, false alarms, missed alarms, explanatory bias, and governance failure. More specifically, the recognition-quality results show that a stable ordinal mapping exists between risk labels and continuous threat scores, supporting an understanding of judgment problems in complex risk analysis as “level allocation after continuous evidence integration” rather than as static, discrete, one-off classification. The system-resilience results further show that different perturbation types are not statistically homogeneous. In particular, fabricated relations, although smaller in overall scale, exhibit stronger structural concentration within the high-risk composition. This means that key vulnerabilities in complex risk information analysis systems do not always stem from the most frequent disturbances, but often from structural distortions that can alter the interpretive direction of event chains or chains of responsibility. Governance-compatibility results further indicate that audit logs, source traceability, and package-level testing are not external add-on requirements, but prerequisites for the interpretive legitimacy of system outputs.

### 5.2. System Resilience Results and Their Dialog with Existing Research (Revisiting Section 4.3)

Section 4.3 shows that the current sample pool forms a stratified stress environment across four principal distortion pathways—semantic perturbation, prompt injection, high-noise conflict, and fabricated relations—and that fabricated relations. However, smaller in total volume, they exhibit stronger structural concentration in flows toward the high-risk zone. Compared with research on the enabling role of automated analysis, this study does not reduce the value of generative inference, retrieval augmentation, and multi-source automated tools to being merely “faster” or “more accurate”; instead, it emphasizes that they must simultaneously satisfy the joint requirements of recognition quality, system resilience, and governance compatibility. This complements existing work on hallucination, automation bias, and distorted explanations: previous studies reveal failures, whereas the present study places them back into the same workflow and explains the conditions under which they jointly emerge. Compared with swarm-intelligence research, the study does not stop at global optimization, search efficiency, or convergence performance. Still, it translates local perception and threshold propagation in collective vigilance into a structural qualification basis before formal comparison. Compared with artificial immune system research, it does not limit that tradition to anomaly detection. Instead, it situates screening, verification, and memory mechanisms within the chain of recognition, recovery, and auditing.

From the perspective of human–machine collaboration and governance research, the distinctiveness of this study lies in embedding governance compatibility as a design objective rather than treating it as an external rule imposed after deployment, and in expressing it quantitatively through the *GCI*, package-level tests, and audit-log foundations. In this way, explainability, responsibility mapping, and mechanisms of human final adjudication cease to be merely discursive claims and instead become system variables that can enter the results section.

### 5.3. Governance Compatibility Results, Alternative Explanations, and Mechanism Boundaries (Revisiting Section 4.4)

Section 4.4 shows that the current sample pool has passed package-level tests such as schema validity, range checks, utility proxy assessment, and constraint consistency; governance compatibility is therefore not an embellishment outside the method, but a systemic property grounded in source traceability, structural consistency, and rule-based generation processes. The analysis must nevertheless acknowledge that the Chapter 4 results may still admit alternative explanations. For example, part of the current structural advantage may arise from the relative richness of the authentic public sample sources themselves rather than entirely from the contribution of biomimetic mechanisms; the stability of risk stratification may also be influenced in part by label design and synthetic rules; and the high performance of governance compatibility indicators may derive from the recording mechanism itself rather than from the framework’s intrinsic superiority. For this reason, the study treats these findings as establishing the basis for formal comparison rather than as direct evidence of superiority.

Nevertheless, the present evidence more strongly supports the explanatory path proposed here. First, sample qualification, risk stratification, perturbation structure, and governance testing do not constitute independent results; rather, they form a mutually reinforcing closed loop within the same framework. Second, the flow patterns of fabricated relations, high-noise conflicts, and prompt injections align closely with the framework’s biomimetic logic of “early discovery–strong screening–responsibility-chain retention.” Third, clearly specified boundary conditions keep the interpretation restrained, thereby reducing the risk of mistaking sample structure for system superiority. Thus, although future work should add formal baseline comparisons and ablation experiments, the existing results already justify treating the biomimetic collective sensing–immune-inspired verification framework as a system-mechanism scheme with explanatory potential for further validation.

### 5.4. Integrated Governance Implications

From a governance perspective, the most immediate implication of this study is that the reliability of complex risk information analysis systems should not be defined solely by accuracy-type indicators, but by the joint performance of recognition quality, system resilience, and governance compatibility. In high-noise, multi-source, multimodal settings, source traceability and audit logging are not merely compliance add-ons; they are necessary conditions for preventing error diffusion and responsibility drift. At the level of organizational design, this means that source ledgers, audit-log templates, risk-escalation pathway records, and interfaces for human final adjudication should be designed synchronously during the system development stage, rather than added post hoc through external institutions as a passive corrective.

A second implication is that governance should be tied to perturbation structure types. The Chapter 4 results show that different perturbation types enter the high-risk interval through different mechanisms; therefore, governance strategies should not treat all anomalies as homogeneous risks. Fabricated relations, prompt injection, and high-noise conflict correspond to different detection, verification, and review configurations. If governance mechanisms are insensitive to perturbation type, resource allocation can become imbalanced: excessive expenditure may be directed toward low-consequence, high-frequency noise, while high-consequence, low-frequency structural distortion receives an insufficient response.

### 5.5. Boundary Conditions, Limitations, and Future Research

The conclusions of this study apply primarily to open-source, multi-source, multimodal complex risk-information analysis settings that retain human final adjudication and take defensive identification, verification, and governance auditing as their objective. They do not automatically generalize to closed-source environments, fully automated execution systems, or scenarios involving the optimization of real-world attack pathways. At the sample level, the current results rely on the structural anchoring of authentic public-source acquisition links and the auditable extension of rule-based derivative samples; therefore, they do not cover all extreme long-tail events or all linguistic regions. At the results level, Chapter 4 now reports controlled benchmark comparisons, module-ablation results, perturbation-specific robustness results, source-held-out validation, *GCI* sensitivity analysis, statistical tests, and theoretical complexity analysis. These results should be interpreted as controlled-benchmark evidence rather than as proof of full external real-world deployment performance.

Future research may advance along three paths. First, it may incorporate non-biomimetic baselines, ablation designs, and multiple rounds of robustness experiments to complete a formal quantitative comparison of the framework’s superiority. Second, without altering the ethical boundary, it may introduce finer-grained cross-lingual and cross-modal distortion analysis to identify differentiated governance requirements for different interference pathways. Third, subject to formal ethical approval, future work may collect behavioral-layer data to test how human final adjudication, explanation granularity, and audit feedback affect judgment quality, thereby extending the current system-level study into collaborative-level research.

### 5.6. Weaknesses, Trade-Offs, and Deployment Constraints

The proposed framework offers clear advantages in traceability, layered verification, and governance-sensitive evaluation, but these advantages come with trade-offs. Table 18 summarizes the main weaknesses, trade-offs, and mitigation strategies that should guide real-world deployment.

### 5.7. Potential Application Domains in Complex Risk Analysis

The framework is most suitable for domains in which information is multi-source, noisy, time-sensitive, and governance-sensitive, but where final high-consequence action should remain subject to human adjudication. Table 19 identifies representative application areas.

## 6. Conclusions

This study examined whether a biomimetic framework integrating collective sensing with immune-inspired verification could provide a rigorous, explicitly bounded approach for optimizing complex risk-information analysis systems under conditions of high noise, multi-source inputs, multimodality, and adversarial disruption. Its purpose was not to proclaim superiority in advance, but to determine whether a biologically informed architecture had already established the structural empirical basis necessary for formal comparison.

At the heart of the study lies a distinctly biomimetic proposition: that two biologically grounded functional logics—distributed vigilance in collective systems and hierarchical recognition and memory in immune systems—can be translated into a unified analytical architecture for complex risk-information analysis. In biological terms, collective vigilance contributes to local perception, weak-signal accumulation, threshold triggering, and coordinated warning. In contrast, immune recognition supports rapid screening, layered verification, memory retention, and a response upon re-encounter. The significance of this study lies in showing that these are not merely evocative analogies. Rather, they can be rendered as engineering variables, algorithmic rules, and auditable evaluative pathways within the same system framework.

Using a dual-layer data architecture composed of authentic public-source samples and rule-generated derivative synthetic samples, and combining biological-to-engineering mechanism translation, multi-objective optimization, NIST-aligned evaluation, and a governance compatibility index, the study assessed the proposed framework through three complementary lines of evidence: recognition performance, recovery under perturbation, and governance auditing.

The findings support three principal conclusions. First, with respect to recognition quality, risk labels and threat scores now exhibit a stable and measurable graded correspondence, thereby establishing a credible basis for formal comparison. Second, with respect to system resilience, the perturbation architecture encompasses the principal distortion pathways—semantic perturbation, prompt injection, high-noise conflict, and fabricated relations—and therefore provides a sufficiently demanding stress environment for formal assessment. Third, with respect to governance compatibility, NIST-aligned evaluation, package-level validation, and the audit-log foundation have already brought governance variables into a computable evaluative framework, thereby establishing an auditable basis for further comparison.

Taken together, these findings constitute a structural empirical closure rather than a final determination of superiority. In other words, the study shows that the proposed framework has reached the level of methodological maturity required for formal system comparison. However, it does not replace the itemized comparative evidence still needed for definitive claims of outperformance.

From the standpoint of biomimetics, the central contribution of this study is not simply that it borrows biological language, but that it preserves biological function at the level of mechanism. The distributed vigilance found in collective organisms is translated into a system logic of local sensing, escalation, and coordinated warning; the hierarchical recognition-memory logic of immune systems is translated into rapid filtering, deep verification, adaptive correction, and retained response capacity. The value of this translation is that it links early discovery, error suppression, adaptive recovery, and governance accountability within a single evaluative structure. In this sense, the study advances biomimetic optimization not as metaphorical inspiration, but as a testable, auditable, and extensible system-mechanism model.

A second contribution lies in the evaluative standard adopted here. Rather than allowing any single performance metric to stand in for the reliability of a complex analytical system, the study places recognition quality, system resilience, and governance compatibility within the same inferential frame. This yields a more exacting criterion for assessing biomimetic intelligent systems in analytically demanding and governance-sensitive environments. A third contribution lies in the explicit treatment of boundary conditions. The framework is advanced not as a complete or final explanatory solution, but as a disciplined biomimetic model whose validity depends on the conditions under which it is applied, tested, and audited.

The practical implications follow directly from this logic. In open-source, multi-source, and multimodal analytical settings that retain human final adjudication, organizations should establish source ledgers, audit logs, and risk-escalation records to preserve traceability and clarify responsibility mapping. Different perturbation types should not be treated as homogeneous forms of risk, because semantic perturbation, prompt injection, high-noise conflict, and fabricated relations call for differentiated configurations of detection, verification, and review. More broadly, deployment decisions should be guided by the joint performance of recognition quality, resilience, and governance compatibility, rather than by accuracy-type indicators alone.

The conclusions of this study remain subject to clear limits. They apply only to complex risk-information analysis settings that are open-source, multi-source, multimodal, retain human final adjudication, and are oriented toward defensive identification, verification, and governance auditing. Extrapolation beyond these conditions requires caution. Although the revised study reports controlled benchmark comparisons, ablation results, perturbation-specific robustness statistics, source-held-out validation, *GCI* sensitivity analysis, and statistical tests, it does not claim full external real-world validation, human-subject trust-calibration evidence, or fully autonomous high-consequence deployment. Further research should therefore test the framework on complete external public corpora, conduct deployment-level runtime and memory benchmarking, calibrate *GCI* weights through expert elicitation or data-driven learning, and evaluate human trust calibration under formal ethical approval.

Overall, this study shows that a biomimetic collective sensing–immune-inspired verification framework can serve as a rigorous methodological starting point for the next stage of comparative, auditable, and governance-aware research in complex risk-information analysis. Its importance lies not in claiming that the final answer has already been reached, but in demonstrating that a biologically grounded, analytically disciplined, and governance-compatible route towards that answer is now in place. The study’s central empirical achievement is therefore not the premature declaration of outperformance, but the successful conversion of a biomimetic conceptual proposition into a structured, comparable, auditable, and governance-sensitive evidentiary architecture.

In its revised form, the manuscript therefore makes a narrower but stronger claim: SVI-IV is a mathematically specified, algorithmically reproducible, and governance-aware framework evaluated under controlled perturbation conditions. The evidence supports controlled-benchmark feasibility and auditability, while full external real-world validation, human-subject trust-calibration testing, and fully autonomous high-consequence deployment remain outside the evidentiary scope of the present study.

## Figures and Tables

**Table 1:** Summary of Data Sources.

Data Layer	Data Source	Primary Content	Use in This Study	Authenticity and Verifiability
Authentic Public Sample Layer	GDELT 2.0	Global events, mentions, cross-lingual news streams, and event logs	Event-level text, event logs, source heterogeneity, and time-series risk signals	Verifiable via official project and data pages
Authentic Public Sample Layer	CrisisMMD	Crisis tweets, images, and human annotations	Multimodal event units: image–text consistency and conflict testing	Verifiable via the official dataset page
Authentic Public Sample Layer	MAVEN-ERE	Event coreference, temporal, causal, and subevent relations	Relation-level verification and event-chain integrity testing	Verifiable via the formal paper and project page
Authentic Public Sample Layer	MIRACL	Retrieval samples and relevance annotations in 18 languages	Assessment of cross-lingual complexity and retrieval robustness	Verifiable via the formal paper
Derivative Synthetic Sample Layer	Prompt-injection samples	Instruction contamination and contextual steering	Adversarial input testing	Rule-generated from authentic samples; reproducible and reviewable
Derivative Synthetic Sample Layer	Semantic-perturbation samples	Light paraphrasing, tonal steering, and semantic shift	Noise-robustness testing	Rule-generated from authentic samples; reproducible and reviewable
**Derivative Synthetic Sample Layer**	Fabricated-relation samples	Mismatched entity, temporal, and causal relations	Relation-level error-chain testing	Rule-generated from authentic samples; reproducible and reviewable
Derivative Synthetic Sample Layer	High-noise confusion samples	Redundancy, conflict, and multi-source interference	Recovery-rate and threshold-propagation testing	Rule-generated from authentic samples; reproducible and reviewable
System Governance Audit Layer	Audit logs and governance indicators	Source contribution, risk-escalation paths, verification results, grounds for human adjudication, and *GCI*-dimension records	Governance compatibility analysis and responsibility-mapping verification	Automatically generated during system output; reproducible and reviewable

Note. This table distinguishes authentic public samples, derivative synthetic samples, and governance audit materials. It clarifies how source authenticity, perturbation testing, and responsibility mapping are separated in the data architecture.

**Table 2:** Baseline System Specifications.

Baseline Family	Signal Aggregation	Verification Logic	Memory Component	Audit Logging	Expected Limitation
Conventional fusion pipeline	Rule-based or weighted fusion	Single-stage validation	Absent	Minimal process trace	Strong at aggregation, but weak at adaptive recovery and governance retention
RAG/LLM analytical baseline	Retriever plus generative synthesis	Prompt-conditioned checking	Context-window only	Output logs without deep responsibility mapping	Strong at synthesis, weaker under adversarial perturbation and traceability demands
SVI-IV proposed framework	Local sensing plus threshold propagation	Rapid screening plus deep verification	Explicit memory updating	Structured audit-log spine	Designed to couple recognition, resilience, and governance compatibility
Module-reduced: no vigilance	No distributed escalation logic	Verification retained	Optional	Retained	Tests dependence on collective sensing and early warning
Module-reduced: no immune memory	Escalation retained	Verification retained, no adaptive correction	Absent	Retained	Tests dependence on retained response and recovery learning

Note. This table defines the comparison families structurally rather than promotionally. It clarifies which capabilities are present, absent, weakened, or redistributed across the proposed framework and its baselines.

**Table 3:** Research Question, Variable, and Metric Alignment.

Research Question	Core Mechanism	Primary Variables	Principal Metrics	Interpretive Purpose
RQ1 Recognition quality	Collective sensing and threshold propagation	Threat score, label consistency, source heterogeneity, verification intensity	Accuracy, *F*_1_, *FER*, *MDR*	Tests whether layered biomimetic sensing improves discrimination and early warning
RQ2 System resilience	Screening, deep verification, and memory updating	Noise intensity, perturbation type, cross-lingual complexity, recovery path	Recovery rate, degradation slope, re-encounter response	Tests whether the framework suppresses error propagation and restores performance under stress
RQ3 Governance compatibility	Audit logging and responsibility mapping	Transparency, traceability, bias sensitivity, human intervenability, accountability, and clarity	*GCI* and audit-log indicators	Tests whether governance variables become computable rather than merely declarative

Note. This table links the research questions, mechanism claims, variable design, and evaluative indicators. It reduces slippage between conceptual claims and empirical tests.

**Table 4:** Sampling and Stratification Rules.

Sampling Component	Rule	Operational Rationale	Contribution to Comparability
Authentic public layer	Select openly verifiable records from the four named corpora	Preserves source authenticity and documentary traceability	Anchors the study in auditable public material
Derivative synthetic layer	Generate perturbations only from authentic source-linked records	Prevents detached fictional sample construction	Preserves source-to-derivative lineage
Risk stratification	Assign low, medium, and high-risk layers through rule-consistent threat scoring	Creates ordered comparison strata	Supports formal discrimination and boundary analysis
Complexity stratification	Track noise intensity, source heterogeneity, and cross-lingual complexity on ordered scales	Prevents single-condition evaluation	Supports robustness interpretation across conditions
Train/validation/test split	Apply 70/15/15 partitioning at the event-unit level	Prevents leakage across optimization and evaluation stages	Supports replicable experimental partitioning

Note. This table makes the sampling regime explicit without overstating completeness. It emphasizes authenticity, traceability, ordered stress conditions, and non-leaking partition logic.

**Table 5:** Sample Composition Summary.

Sample Block	Count	Share or Split	Interpretive Role
Authentic public samples	7200	60.0% of total pool	Primary empirical anchor for source authenticity and structural realism
Derivative synthetic samples	3000	25.0% of total pool	Stress-testing layer for auditable perturbation conditions
Multimodal composite samples	1800	15.0% of total pool	Additional burden for cross-modal consistency testing
Train/validation/test split	8400/1800/1800	70%/15%/15%	Partition regime for optimization and evaluation

Note. This table consolidates the sample architecture into a single place. It shows how the total pool is layered and why each block matters analytically.

**Table 6:** Core Experimental Configuration.

Configuration Element	Specification	Why It Matters
Software stack	Python 3.11; PyTorch 2.3; Transformers 4.44; scikit-learn 1.5; pandas 2.2; statsmodels 0.14	Defines the executable environment
Hardware	Ubuntu 22.04; NVIDIA A100 80 GB; 256 GB RAM; 32-core CPU	Defines computational comparability
Total sample pool	12,000 event-level units	Defines the analytical scale
Partitioning	70% training; 15% validation; 15% test	Defines non-leaking evaluation structure
Repetition regime	10 routine runs; 5 ablation runs; 5 robustness runs	Improves stability of estimation
Random seeds	{7, 11, 29, 41, 57, 73, 89, 101, 131, 151}	Supports replication of stochastic procedures
Threshold search	Grid search over vigilance cutoffs and memory-update interval 0.1–0.5	Makes optimization decisions inspectable

Note. This table gathers the configuration elements required for replication. It reduces ambiguity about environment, scale, repetition, random seeds, and threshold-search settings.

**Table 7:** Audit-Log Schema and Reproducibility Materials.

Item	Illustrative Content	Reproducibility Function
event_id	Event-level unique identifier	Preserves record-level traceability
source_id and source ledger link	Corpus, access point, version, checksum	Links outputs back to public-source provenance
perturbation_type	Prompt injection, semantic shift, fabricated relation, high-noise conflict	Makes stress condition auditable
verification_outcome	Rapid-screen, deep-check, escalation result	Documents a layered decision path
human_review_flag	Indicator for retained human adjudication	Preserves governance boundary
configuration bundle	Seed, threshold set, model version, run identifier	Makes runs reproducible
Public materials	Dataset record, parameter files, templates, schema tables	Supports public re-use and scrutiny

Note. This table links governance evidence with executable replication. It shows how lineage, configuration, verification outcomes, and human-review boundaries are recorded.

**Table 8:** Stratified Statistics of Risk Level and Threat Score.

Risk Level	Sample Size	Mean	Standard Deviation	Minimum–Maximum
low	1149	49.18	13.88	8.03–90.02
medium	1139	54.71	14.24	9.73–93.45
high	712	60.60	13.21	17.36–96.64

Note. This table reports sample size and threat-score distribution across risk levels. It supports the measurement basis of RQ1 by showing a stable upward gradient from low to high risk.

**Table 9:** Summary of Package-Level Validation Results for the Sample Pool.

Dimension	Indicator	Result	Status
schema_validity	overall_missing_rate	0.0	pass
range_check	noise_strength	(1, 5)	pass
range_check	crosslingual_complexity	(1, 5)	pass
range_check	source_heterogeneity	(1, 5)	pass
range_check	threat_score	(8.03, 96.64)	pass
utility_proxy	risk_label_mean_threat_score	high = 60.60 low = 49.18 medium = 54.71	pass
constraint	review_flag_consistency	1	pass

Note. This table summarizes the package-level validation items used in the results section. It indicates structural completeness, controlled range, discriminable risk stratification, and rule consistency, without claiming full external-corpus validation.

**Table 10:** Alignment of Research Questions, Evidentiary Paths, and Current Result Status.

Research Question	Evidentiary Path	Key Indicators	Current Result Status	Degree of Support
RQ1	Recognition Quality	Risk stratification, threat scores, label consistency, and controlled benchmark performance	Controlled benchmark comparison reported in Table 11	Supported within the controlled benchmark
RQ2	System Resilience	Perturbation structure, recovery proxy, module ablation, and source-held-out stability	Perturbation-specific robustness and ablation evidence reported in Table 12, Table 13 and Table 14	Supported within the controlled benchmark
RQ3	Governance Compatibility	*GCI*, *GCI* sensitivity, audit-log foundations, and package-level validation	Computable governance evaluation and sensitivity analysis reported	Supported within the controlled benchmark

Note. This table distinguishes what is already supported by measured structural evidence from what still requires itemized comparative testing. It functions as a claim-discipline device.

**Table 11:** Comparative Performance Results Across Baselines and SVI-IV.

*GCI*	Recovery	*AUC*	*MDR*	*FER*	*F*_1_	Accuracy	Method
57.25	0.998	0.647	0.022	0.919	0.767	0.634	Threat threshold
64.00	1.000	0.586	0.027	0.933	0.762	0.626	Rule fusion
62.50	1.000	0.703	0.072	0.768	0.772	0.661	Logistic
56.00	1.000	0.652	0.130	0.725	0.750	0.642	Random forest
56.40	0.979	0.697	0.139	0.652	0.760	0.664	Gradient boosting
86.45	1.000	0.697	0.119	0.661	0.769	0.673	SVI-IV
52.50	1.000	0.580	0.040	0.957	0.752	0.609	Isolation Forest

Note. This table reports controlled benchmark results using the 3000-sample derivative perturbation dataset. The values are not presented as full external deployment results.

**Table 12:** Module-Ablation Results for SVI-IV.

Δ*GCI*	*AUC*	*MDR*	*FER*	Δ*F*_1_	*F*_1_	Accuracy	Variant
Reference	0.697	0.119	0.661	Reference	0.769	0.673	Full SVI-IV
−5.0	0.679	0.142	0.652	−0.011	0.758	0.662	No sensing
−5.2	0.690	0.137	0.655	−0.009	0.760	0.664	No immune
−3.8	0.694	0.155	0.609	−0.009	0.760	0.671	No memory
−10.5	0.692	0.182	0.588	−0.020	0.749	0.662	No audit

Note. This table reports module-ablation results under controlled benchmark conditions. Delta values are calculated relative to the full SVI-IV variant; the full model is therefore marked as the reference case.

**Table 13:** Perturbation-Specific Robustness Results for the SVI-IV Controlled Model.

*AUC*	*MDR*	*FER*	*F*_1_	Accuracy	N	Perturbation Type
0.633	0.085	0.750	0.740	0.631	225	high_noise_conflict
0.666	0.133	0.703	0.777	0.673	217	prompt_injection
0.698	0.149	0.676	0.757	0.656	192	relation_fabrication
0.769	0.111	0.538	0.796	0.722	266	semantic_perturbation

Note. This table reports perturbation-specific controlled benchmark performance. It does not claim to cover all real-world perturbations.

**Table 14:** Source-Held-Out Validation Within the Controlled Derivative Benchmark.

Recovery	*AUC*	*MDR*	*FER*	*F*_1_	Accuracy	N	Held-Out Source
1.000	0.733	0.108	0.606	0.769	0.686	602	CrisisMMD
1.000	0.702	0.211	0.518	0.755	0.676	1355	GDELT
1.000	0.697	0.203	0.601	0.735	0.645	583	MAVEN-ERE
0.973	0.771	0.071	0.616	0.803	0.720	460	MIRACL

Note. Source-held-out validation tests the generalization across source families inside the derivative benchmark. It is not presented as full external real-world validation.

**Table 15:** *GCI* Sensitivity Under Alternative Weighting Schemes.

Accountability-Prioritized	Human-Intervention-Prioritized	Traceability-Prioritized	Transparency-Prioritized	Equal Weight	Original	Method
55.80	56.90	57.90	58.00	56.00	57.25	Threat-score threshold
62.95	63.50	64.50	64.65	63.00	64.00	Rule-based fusion
61.65	62.20	63.20	62.55	62.00	62.50	Logistic regression
55.40	55.40	56.40	55.40	56.00	56.00	Random forest
55.76	55.76	56.76	55.76	56.40	56.40	Gradient boosting
51.70	51.70	52.70	52.50	52.00	52.50	Isolation Forest
86.65	86.32	86.82	86.35	86.60	86.45	SVI-IV controlled model

Note. *GCI* scores are design-based governance indicators; the sensitivity analysis tests whether the governance ranking depends on a specific weighting scheme.

**Table 16:** Bootstrap Statistical Testing for SVI-IV Versus Baselines.

Adjusted *p*-Value	*p*-Value	95% CI	Mean Difference	Metric	Comparison
*p* < 0.001	*p* < 0.001	[0.016, 0.078]	0.048	Accuracy	SVI-IV vs. Rule-based fusion
*p* < 0.001	*p* < 0.001	[0.006, 0.058]	0.031	Accuracy	SVI-IV vs. Random forest
*p* < 0.001	*p* < 0.001	[0.033, 0.093]	0.063	Accuracy	SVI-IV vs. Isolation Forest
0.020	0.007	[0.013, 0.069]	0.039	Accuracy	SVI-IV vs. Threat-score threshold
0.112	0.047	[0.002, 0.037]	0.019	*F*_1_	SVI-IV vs. Random forest
0.200	0.100	[−0.003, 0.037]	0.017	*F*_1_	SVI-IV vs. Isolation Forest
0.389	0.227	[−0.006, 0.021]	0.008	*F*_1_	SVI-IV vs. Gradient boosting
0.450	0.300	[−0.011, 0.036]	0.012	Accuracy	SVI-IV vs. Logistic regression
0.684	0.513	[−0.013, 0.027]	0.007	*F*_1_	SVI-IV vs. Rule-based fusion
0.616	0.513	[−0.011, 0.025]	0.007	Accuracy	SVI-IV vs. Gradient boosting
0.785	0.720	[−0.018, 0.013]	−0.003	*F*_1_	SVI-IV vs. Logistic regression
0.987	0.987	[−0.018, 0.020]	0.001	*F*_1_	SVI-IV vs. Threat-score threshold

Note. Differences are computed as SVI-IV minus the comparator. Adjusted *p*-values use the Benjamini–Hochberg correction across reported comparisons.

**Table 17:** Computational Complexity and Scalability Profile.

Main Cost Driver	Space Complexity	Time Complexity	Module
Number of source and categorical fields	*O*(*nm*)	*O*(*nm*)	Source and feature encoding
Feature dimension and sample size	*O*(*nd*)	*O*(*nd*)	Collective sensing
Number of event-level samples	*O*(*n*)	*O*(*n*)	Threshold escalation
Number of verification indicators	*O*(*k*)	*O*(*nk*)	Immune verification
Perturbation-memory states	*O*(*r*)	O(*nr*)	Memory updating
Governance dimensions	*O*(*ng*)	*O*(*ng*)	*GCI* computation
Audit-log fields	*O*(*na*)	*O*(*na*)	Audit-log generation

Note. *n* denotes samples, *m* source fields, *d* feature dimension, *k* verification indicators, *r* memory states, *g* governance dimensions, and *a* audit-log fields.

**Table 18:** Advantages, Trade-Offs, and Mitigation Strategies.

Mitigation Strategy	Corresponding Trade-Off	Advantage
Selective logging and log compression	Larger audit-log burden	Stronger traceability
Risk-based escalation and staged checking	Higher latency	Deeper verification
Sensitivity analysis and fixed configuration records	Additional parameters and thresholds	Perturbation robustness
Tiered automation with retained human override	Lower full automation in high-consequence settings	Human final adjudication
Source-held-out validation and future full external validation	External validity limits	Synthetic perturbation control
Transparent *GCI* weights and sensitivity testing	More complex evaluation protocol	Governance-aware design

Note. The table makes explicit that the framework is not cost-free; its governance and verification gains require computational, operational, and validation discipline.

**Table 19:** Potential Application Domains for SVI-IV.

Governance Requirement	Relevant SVI-IV Modules	Risk Signals	Application Domain
Source traceability and escalation logs	Collective sensing; memory updating	Supplier disruption, port delay, sanctions, logistics anomalies	Supply chain disruption monitoring
Accountability and intervention records	Threshold escalation; human adjudication	Weather, delay, aircraft status, passenger-flow disruption	Flight planning and passenger re-accommodation
Intervention logging and false-alarm control	Local sensing; rapid screening	Accidents, congestion, and infrastructure failure	Transportation network risk analysis
Rumor control and source accountability	Immune verification; audit logs	Multilingual social media, images, and conflicting reports	Crisis-response information triage
Audit trail and human override	Rapid screening; deep verification	Prompt injection, noisy logs, suspicious relations	Cybersecurity triage

Note. These domains are illustrative. The present study supports the feasibility of controlled benchmarking, while full-domain deployment requires external validation and domain-specific governance review.

## Data Availability

The authentic public sample sources used in this study include GDELT 2.0, CrisisMMD, MAVEN-ERE, and MIRACL. The derivative synthetic samples generated from these public data using rule-based procedures, together with parameter configuration files, the random-seed set, perturbation templates, and the audit-log schema, are publicly documented in the Harvard Dataverse record: MENG, WEI [29]. “Collective Vigilance Intelligence: A Research Dataset for a Bionic Collective Perception-Immune Verification Optimization Framework for Complex Risk Information Analysis.” https://doi.org/10.7910/DVN/HKN1ED, Harvard Dataverse, V1. The Dataverse record provides the public reference point for lineage verification. At the same time, the present manuscript specifies the methodological rules required to reproduce the analytical workflow from source-layer construction to governance-audit output. The uploaded data package also provides official acquisition links and scripts for public-source datasets; however, the full external corpora are not mirrored in this manuscript package. Full NIST-style cross-layer utility, fidelity, and privacy validation, therefore, requires direct acquisition of public corpora from their official sources and comparison with the synthetic layer.
